# Signal Peptide Cleavage from GP5 of PRRSV: A Minor Fraction of Molecules Retains the Decoy Epitope, a Presumed Molecular Cause for Viral Persistence

**DOI:** 10.1371/journal.pone.0065548

**Published:** 2013-06-06

**Authors:** Bastian Thaa, Balaji Chandrasekhar Sinhadri, Claudia Tielesch, Eberhard Krause, Michael Veit

**Affiliations:** 1 Institute of Virology, Department of Veterinary Medicine, Free University Berlin, Berlin, Germany; 2 Leibniz Institute of Molecular Pharmacology (FMP), Berlin, Germany; Nanyang Technological University, Singapore

## Abstract

Porcine reproductive and respiratory syndrome virus (PRRSV) is the major pathogen in the pig industry. Variability of the antigens and persistence are the biggest challenges for successful control and elimination of the disease. GP5, the major glycoprotein of PRRSV, is considered an important target of neutralizing antibodies, which however appear only late in infection. This was attributed to the presence of a “decoy epitope” located near a hypervariable region of GP5. This region also harbors the predicted signal peptide cleavage sites and (dependent on the virus strain) a variable number of potential *N*-glycosylation sites. Molecular processing of GP5 has not been addressed experimentally so far: whether and where the signal peptide is cleaved and (as a consequence) whether the “decoy epitope” is present in virus particles. We show that the signal peptide of GP5 from the American type 2 reference strain VR-2332 is cleaved, both during *in vitro* translation in the presence of microsomes and in transfected cells. This was found to be independent of neighboring glycosylation sites and occurred in a variety of porcine cells for GP5 sequences derived from various type 2 strains. The exact signal peptide cleavage site was elucidated by mass spectrometry of virus-derived and recombinant GP5. The results revealed that the signal peptide of GP5 is cleaved at two sites. As a result, a mixture of GP5 proteins exists in virus particles, some of which still contain the “decoy epitope” sequence. Heterogeneity was also observed for the use of glycosylation sites in the hypervariable region. Lastly, GP5 mutants were engineered where one of the signal peptide cleavage sites was blocked. Wildtype GP5 exhibited exactly the same SDS-PAGE mobility as the mutant that is cleavable at site 2 only. This indicates that the overwhelming majority of all GP5 molecules does not contain the “decoy epitope”.

## Introduction

Porcine reproductive and respiratory syndrome virus (PRRSV) is one of the most important swine pathogens, causing enormous economic losses. PRRSV is an enveloped virus and belongs to the family *Arteriviridae* in the order *Nidovirales*, together with equine arteritis virus (EAV), murine lactate dehydrogenase-elevating virus (LDV) and simian haemorrhagic fever virus (SHFV) [1]. Originally, distinct genotypes were identified in Europe (type 1, prototype: Lelystad virus, [2]) and North America (type 2, reference strain: VR-2332, [3]) in the early 1990s. Meanwhile, these viruses have spread worldwide, involving also the emergence of highly virulent, type 2-related PRRSV in Asia since 2006 [4].

The positive-sense RNA genome of PRRSV encompasses approximately 15 kb and contains a set of nested open reading frames (ORF). Of these, ORF2–7 encode structural proteins of the virus. The glycoproteins (GP) 2, 3, and 4 (expressed from ORF2, 3, and 4, respectively) form a heterotrimeric complex in the membrane of the mature virus and are important for cell tropism [5], [6] and virus entry/uncoating by interaction with the essential receptor CD163 [7]. ORF5 and 6 code for GP5 and M, respectively, and form a heterodimer, probably by disulfide bond formation [8], which is the major component of the viral envelope. ORF7 encodes the nucleocapsid (N) protein, which complexes the viral genome in the mature virion. There are two more proteins, encoded by alternative reading frames in ORF2 (ORF2b, encoding the envelope (E) protein) and ORF5 (ORF5a). All structural proteins (possibly with the exception of the recently discovered ORF5a protein [9], [10]) are essential for infectivity. The GP2/3/4 complex and E are dispensable for particle formation, while GP5/M and N are absolutely required for assembly and budding [11]. The major glycoprotein complex GP5/M is also involved in virus entry by binding to the virus receptors heparansulfate and sialoadhesin (CD169), mediating virus attachment and receptor-mediated endocytosis [12].

The virus has a restricted cell tropism *in vitro*. In animals, it enters and replicates in porcine alveolar macrophages, viral antigen has also been detected in resident macrophages of various lymphoid tissues as well as in other cell types [13]. Clinical symptoms, appearing early after infection, are mainly respiratory in growing pigs and promote the manifestation of the “Porcine Respiratory Disease Complex” (multifactorial respiratory disease). In pregnant sows, infection often leads to reproductive failure (abortion, premature farrowing). In neonatal piglets, mortality rates are high [14], [15].

Viremia is sustained for up to four weeks after infection. Beyond that phase, however, the virus is typically not cleared from the body, but is persistently present at a continuous low level of replication, predominantly in lymphoid tissues, and continues to be shed. Only after 4–6 months will the virus be cleared completely from the body [16], [17].

It is generally assumed that the host’s immune system is incapable of setting up a robust immune response against the virus, leading to this persistence phenomenon [18]. While there is a strong antibody response directed against N and GP5 few days after infection, these antibodies do not neutralize the virus [19]. Neutralizing antibodies, however, appear only late, after more than four weeks after infection [19]. Their appearance coincides with clearing of virus from blood. GP5 is considered one major target of neutralizing antibodies [20], [21], albeit not in all reports [22]. Neutralizing epitopes were also described and mapped in other PRRSV proteins, notably M [23] and – at least for the European genotype 1– GP4 [24]. The importance of neutralizing antibodies is reflected by the findings that serum from convalescent pigs [25] and passive transfer of neutralizing antibodies [26] both provide protection to homologous challenge and clear virus from blood.

**Figure 1:**
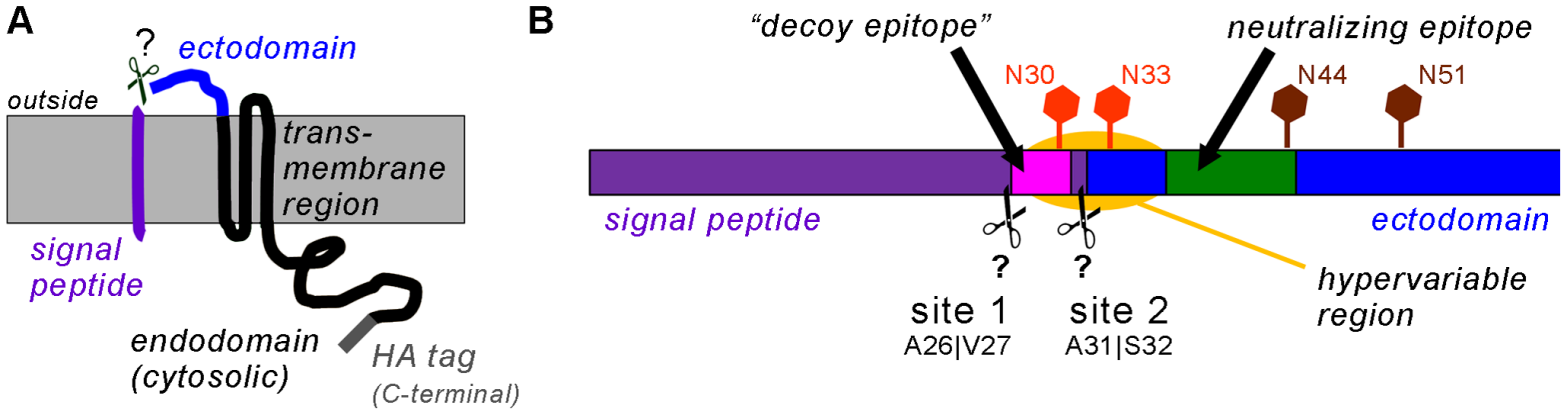
Schematic representation of PRRSV GP5 and its possible processing. (A), Topology of unprocessed PRRSV GP5 with signal peptide (purple, ∼30 amino acids), ectodomain (blue, ∼30 residues), a hydrophobic transmembrane stretch (∼70 amino acids, black) and a cytosolic/virus-internal endodomain (∼70 residues, black). The position of the *C*-terminal HA tag used in this study is indicated (dark grey). Membrane in light grey. (B), Signal peptide cleavage is predicted to occur at two sites: either at A26|V27 (site 1) or A31|S32 (site 2) as indicated. The neutralizing epitope (green) is present in all predicted variants, but the “decoy epitope” (magenta) is not preserved in the mature protein by cleavage at site 2. However, bioinformatic prediction tools rely on the amino acid sequence alone and do not take into account possible carbohydrate attachment to non-conserved glycosylation sites (red) located in the hypervariable region (yellow) and to conserved glycosylation sites (brown). The situation for PRRSV type 2 reference strain VR-2332 is depicted.

GP5 encompasses 200 amino acids (in the North American genotype 2) and comprises an *N*-terminal signal peptide directing protein synthesis to the rough endoplasmic reticulum (ER), followed by an ectodomain of roughly 30 amino acids, containing several *N*-glycosylation sites, two of which (N44 and N51) are highly conserved between GP5 of virus strains. The region between residues 63 and 135 is hydrophobic and assumed to span the membrane three times. The *C*-terminal part (135–200) is most probably located in the cytosol, ending up in the virus interior. See Fig. 1A for a topology sketch of the protein.

GP5 is not only the most variable structural protein of PRRSV, but even one of the most variable proteins of all viruses [27]. This explains why the currently available vaccines protect against a homologous, but not a heterologous infection. Of note, there is a hypervariable region at the border between signal peptide and ectodomain. Since this region is rich in serine and asparagine codons (AGC and AAC, respectively), this often leads to the addition or loss of *N*-glycosylation sites (consensus sequence: N-X-S/T). Serial passaging of virus (PRRSV type 2 reference strain VR-2332) between pigs resulted in numerous mutations in GP5, including the changes N33S and D34N, removing and adding potential glycosylation sites, respectively [28]. The mutation D34N was also observed during the course of infection when the viral sequences in individual pigs were followed after experimental infection with PRRSV VR-2332 for 132 days [29].

Besides variability of the main antigen GP5, persistence of PRRSV poses the biggest challenge for the successful control and elimination of the disease. Several hypotheses about the mechanistic basis for persistence have been put forward (for review, see [30], [31]). Here, the focus shall be on the molecular requirements for one of these controversially discussed hypotheses, the “decoy epitope” hypothesis by Lopez and Osorio [32].

In GP5 of type 2 PRRSV (reference strain: VR-2332), a neutralizing epitope was determined by Pepscan analysis [33] and phage display [34] to comprise amino acids 37–44 (in the ectodomain). This epitope was termed “epitope B” since another epitope further upstream (residues 27–31, “epitope A”) was identified as well [34]. Epitope A elicits an early and strong, but non-neutralizing antibody response, while epitope B appears to be less immunogenic and induces a neutralizing antibody response only late (see Fig. 1B). Thus, the hypothesis was put forward by Lopez and Osorio that epitope A might work as a “decoy epitope” [32]. Decoy epitopes are non-neutralizing, immunodominant epitopes which, when present, decrease the induction or reactivity of antibodies against a nearby neutralizing epitope – a mode of action that was shown to occur e.g. in GP41 of human immunodeficiency virus [35]. For PRRSV of the European genotype (type 1), a “decoy epitope” could not be identified so far, but a neutralizing epitope was described [36].

The “decoy epitope” hypothesis makes the following predictions: Initially after infection of pigs the “decoy epitope” must be present in fully processed GP5. However, this epitope is situated in (or near) the signal peptide. Thus, the extent and exact position of signal peptide cleavage critically determines whether the “decoy epitope” is present in mature GP5. Since peptides covering the complete (unprocessed) protein sequence were used for identification of epitopes, it is not known whether the identified sequences are present in mature GP5.

Later during replication of PRRSV, the “decoy epitope” must be eliminated such that a neutralizing antibody response against epitope B can be raised that may help to clear PRRSV from the body. Elimination of the “decoy epitope” might be achieved by mutations in the hypervariable region that affect signal peptide cleavage. To allow for signal peptide cleavage, the residues at positions –3 and –1 with respect to the cleavage site have to be small and uncharged, e.g. alanines [37], and point mutations in this region might create or destroy a cleavage site. In addition, acquisition or shifting of glycosylation sites could be crucial in this respect: Since both the initial core glycosylation and signal peptide cleavage occur co-translationally by the ER-resident oligosaccharyl transferase and signal peptidase, respectively [37], [38], these simultaneous processes could influence each other: Glycosylation might interfere with signal peptide cleavage since the presence of a bulky glycan structure might prevent accessibility of signal peptidase to a cleavage site that would be suitable in principle. Also, different porcine cells might process GP5 differentially such that the signal peptide (including the “decoy epitope”) is removed in cells that are infected late in the course of infection.

Although the “decoy epitope” hypothesis makes precise predictions regarding the primary structure of GP5, none of the molecular requirements have been analyzed by biochemical means. We therefore assessed experimentally whether and where the signal peptide of GP5 is cleaved and whether this is influenced by the presence or absence of glycans near the cleavage site.

## Results

Signal peptide cleavage is not governed by a consensus sequence like, for instance, *N*-glycosylation, but can be predicted bioinformatically using SignalP 4.0 (www.cbs.dtu.dk/services/SignalP/, [39]). We applied this tool to predict whether the signal peptide is cleaved from GP5 proteins of the different *Arterivirus* species. Surprisingly, very different results regarding the probability of cleavage and the location of the cleavage site were obtained (Table 1). Whereas GP5 of EAV is predicted to contain a usual, short signal peptide (18 amino acids), which is cleaved with high confidence (D score of 0.91), the signal peptide of SHFV-GP5 is longer (41 amino acids) and predicted not to be cleaved. A value of 0.34 was calculated for the D score, which is below the threshold for cleavage of 0.45. An intermediate D score of 0.64 was obtained for cleavage of GP5 from LDV, and the values are 0.85 and 0.76 for GP5 from the reference strains of PRRSV type 1 and 2, respectively.

**Table 1:** Signal peptide cleavage prediction for GP5 of all Arteriviruses.

Virus	N-terminal amino acid sequence (1–50)	D
**EAV**	mlsmivllfllwgapsha|YFSYYTAQRFTDFTLCMLTDRGVIANLLRYDE…	0.91
**PRRSV 1**	mrcshklgrfltphscfwwlfllctglswsfa|DGNGDSSTYQYIY**N**LTIC…	0.85
**PRRSV 2**	mlekcltagccsrllslwcivpfcfa|*vla* ***n***a|S**N**DSSSHLQLIY**N**LTLCEL…	0.76
**LDV**	mkclkklgsgwipsrllpfcfilyflstenacaa|G**N**SSTKNLIY**N**LTLCE…	0.61
**SHFV**	MylclgrsetpliglfrtsstsiswfyvlffvsitfsstgaSE**N**NTGTTW…	(0.34)

Representative *N*-terminal sequences (1–50) of GP5 of each member of the *Arterivirus* genus: EAV, equine arteritis virus (strain Bucyrus, GenBank accession number [ABI64076.1], PRRSV: porcine reproductive and respiratory syndrome virus (type 1/European, strain Lelystad [AAA46278.1] and type 2/North American, strain VR 2332 [AAD12129.1]), LDV: murine lactate dehydrogenase-elevating virus (NCBI reference sequence [NP_042577.1]), SHFV: simian hemorrhagic fever virus (NCBI reference sequence [NP_203550.1]). The predicted signal peptide (in small letters) and the “D value” for the most probable cleavage site (vertical bar) according to bioinformatics prediction with SignalP 4.0 are indicated. (D is a measure for cleavage likelihood, threshold: 0.45– note that the signal peptide of SHFV is predicted not to be cleaved.) Potential *N*-glycosylation sites are highlighted in bold (proven experimentally for LDV [57]). For PRRSV type 2, two signal peptide cleavage sites are possible (indicated with vertical bar). The decoy epitope (*VLAN*, in italics) would be absent in the mature protein if cleavage is performed at the most likely site (A31|S32).

In addition, two different cleavage sites are possible for the North American (type 2) PRRSV (reference strain VR-2332). Apart from the most probable cleavage site (A31|S32, here designated “site 2”), cleavage could also occur further upstream, between alanine 26 and valine 27 (“site 1”). While SignalP 4.0 provides the D score for the most probable cleavage position only, alternative possible cleavage sites can be considered by the “Y score”, which is reported for every residue. This score is 0.72 for site 2 and slightly lower (0.667) for site 1 (see Table S1). Intriguingly, the mature GP5 would be devoid of the “decoy epitope” if signal peptide cleavage occurred at site 2, but the sequence would be present upon cleavage at site 1 or if the signal peptide remained uncleaved.

We consider it unlikely that the homologous protein from different viruses of the same family is cleaved in some species and not cleaved in others since this would generate proteins with very different membrane topologies, which are unlikely to have an identical function. Hence, SignalP 4.0 (despite its confidence of around 90% [39]) might yield inaccurate results in the case of Arterivirus GP5. Also, this prediction tool does not take into account the potential use of glycosylation sites located near the potential cleavage site(s). Glycosylation of these residues might interfere with signal peptide cleavage since the presence of a bulky glycan structure could prevent access of signal peptidase to a cleavage site that would be suitable in principle.

### Analyzing Signal Peptide Cleavage of GP5 *in vitro* using Porcine Microsomes

We aimed at deciphering experimentally whether the signal peptide of GP5 is cleaved and whether this is influenced by glycans near the signal peptide cleavage site. To this end, we first employed *in vitro* transcription/translation/translocation, the classical method to analyze signal peptide cleavage in ER-directed membrane proteins [40]. In this cell-free assay, the gene of interest is transcribed into RNA and translated into (unmodified) protein. Signal peptide processing and glycosylation can only occur upon supplying microsomal membranes (biochemical preparations of ER/Golgi). By comparing protein sizes generated in the absence and presence of microsomal membranes, conclusions can be drawn on protein processing.

The open reading frame (ORF) encoding GP5 (strain VR-2332) was cloned into the plasmid pCMV-TnT, including a *C*-terminal HA tag to enable detection of the protein by Western blot. Based on this construct (GP5–HA wt), a set of mutants was generated in which the potential glycosylation sites N30 and N33 near the predicted signal peptide cleavage position were replaced by serines, individually or in combination (resulting in the mutants GP5–HA N30S, GP5–HA N33S and GP5–HA N30S, N33S). Note that such variation has been described for natural isolates [41]. The other two potential glycosylation sites (N44 and N51) were left unchanged. There are no significant differences in the parameters of signal peptide cleavage prediction as analyzed with SignalP 4.0 between GP5–HA wt and the mutants thereof (see Table S1). Note that glycans are not considered by these predictions.

**Figure 2:**
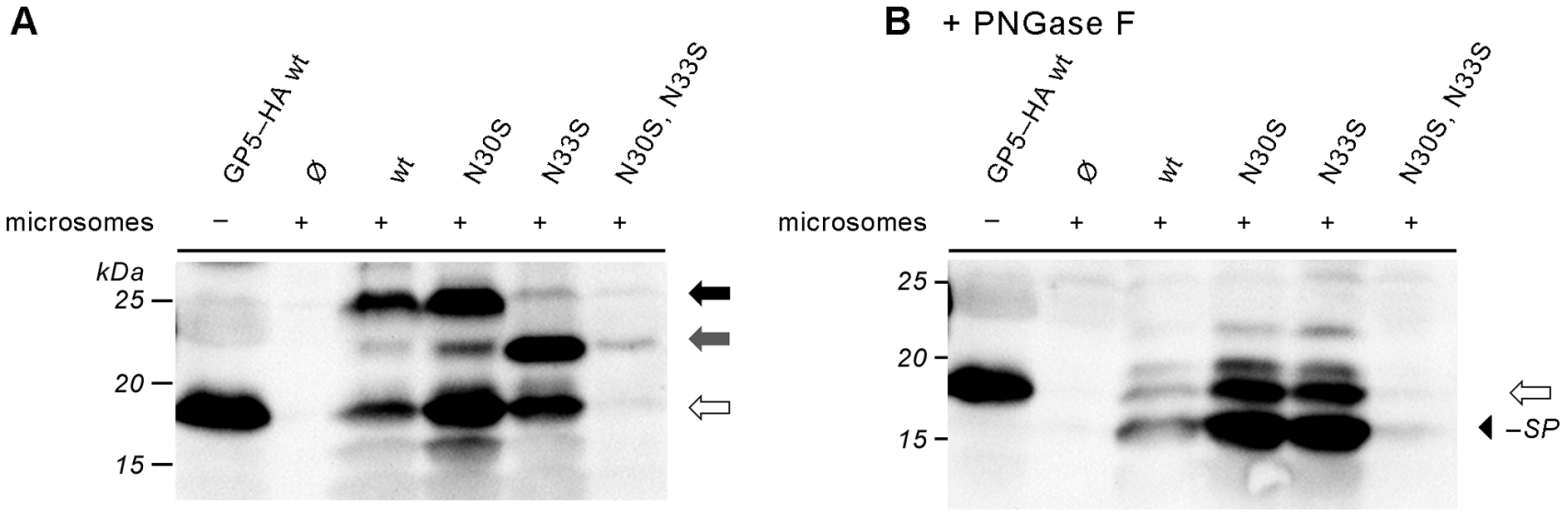
*in vitro*-transcription/translation of GP5–HA to assess processing (glycosylation/signal peptide cleavage). (A), Plasmids encoding GP5–HA was subjected to *in vitro*-transcription/translation with rabbit reticulocyte lysate in the absence (–) or presence (+) of porcine pancreatic microsomes. The products were analysed by SDS-PAGE and Western blot (anti-HA tag). Wildtype (wt) and mutants with deleted or added glycosylation sites near the signal peptide cleavage site were employed; Ø, empty plasmid control. Molecular weight marker is indicated on the left-hand side, arrows on the right-hand side show the positions of unprocessed GP5–HA (white), fully glycosylated GP5–HA (black), and GP5–HA lacking one glycan (grey). (B), Glycans from the products of (A) were removed with PNGase F prior to SDS-PAGE and Western blot. Deglycosylated protein lacking the signal peptide (black arrowhead) is smaller than unprocessed GP5–HA and deglycosylated protein containing the signal peptide (white arrow), indicating signal peptide cleavage.

These constructs were used for *in vitro* transcription/translation followed by SDS-PAGE and Western blot. When the plasmid encoding the wildtype (wt) sequence of GP5 with HA tag was employed, a protein with the apparent molecular mass of 19 kDa was produced (Fig. 2A, leftmost lane). This is smaller than calculated from the amino acid sequence of GP5–HA with signal peptide (23.5 kDa), but specific as evidenced by a control reaction using empty vector (Fig. 2A, lane 2). Thus, due to this aberrant SDS-PAGE mobility of GP5, conclusions regarding signal peptide cleavage cannot be drawn by simply comparing the observed with the predicted molecular weight.

To achieve processing of the protein, we prepared microsomes from the pancreas of a pig, the natural host of PRRSV. Upon *in vitro* transcription/translation of GP5–HA wt in the presence of these microsomes, an additional 26-kDa band appeared (Fig. 2A, third lane), indicating that GP5 was translocated into the lumen of the ER, where it was glycosylated. Since protein translocation *in vitro* is never perfectly efficient, a subfraction of GP5–HA was still present in the unprocessed form as evidenced by the 19-kDa band.

In addition, another (albeit weak) band at around 23 kDa can be discerned. As one glycan typically accounts for approximately 2.5 kDa [42], this subfraction of GP5 most likely lacks one carbohydrate chain.

When GP5–HA N30S was made in the presence of the porcine microsomes (lane 4), the same major band at 26 kDa was seen. Since the removal of the glycosylation site N30 did not reduce the electrophoretic mobility of the protein, this site is either not used or not present in the processed protein (due to cleavage of the signal peptide).

In contrast, removal of the glycosylation site at position 33 (GP5–HA N33S, lane 5) reduced the molecular weight of the expressed protein to around 23 kDa. Thus, N33 is used as a glycosylation site in GP5. The mutant GP5–HA N30S, N33S (with additional replacement of the N30 glycosylation site, lane 6), albeit only poorly expressed or unstable under the experimental conditions, ran like the N33S mutant in SDS-PAGE. – Overall, the band pattern indicates that N33, but not N30, is used as a glycosylation site in the vast majority of GP5 molecules.

To assess whether the signal peptide had been removed during processing of GP5–HA in the presence of microsomes, the *N*-linked glycosylations were removed from the proteins by treating the samples with peptide-*N*-glycosidase F (PNGase F). Deglycosylated GP5–HA should have the same size as GP5–HA synthesized in the absence of microsomes if the signal peptide is not cleaved, but would be smaller by 3 kDa if the signal peptide is absent in the mature protein. When GP5–HA produced with microsomes was digested with PNGase F, a major band at 16 kDa appeared (Fig. 2B, lane 3), which runs well below GP5–HA produced without microsomes (lane 1), indicating that the signal peptide was cleaved. Yet, the 19-kDa band remained unchanged by this treatment, yielding further evidence that it corresponds to unprocessed protein. When the GP5–HA glycosylation mutants were deglycosylated with PNGase F, the same band pattern as for GP5–HA wt was seen (Fig. 2B; the weak bands above 19 kDa are probably due to incomplete PNGase F digestion). Thus, removal of neighboring glycosylation sites does not affect signal peptide cleavage, neither qualitatively nor quantitatively. However, it is difficult to precisely assess the efficiency of signal peptide cleavage since translocation and glycosylation are not 100% efficient and also vary between microsome preparations.

### Analyzing Signal Peptide Cleavage of GP5 in Transfected Cells

To overcome these technical limitations of *in vitro* transcription/translation, the GP5–HA variants were expressed in cells, where authentic processing of the protein can be expected. In addition to the glycosylation mutants described above, another mutant (GP5–HA D34N) was generated by introduction of a third potential glycosylation site near the signal peptide cleavage site. This amino acid exchange in GP5 was observed in the course of experimental infection of pigs [29].

**Figure 3:**
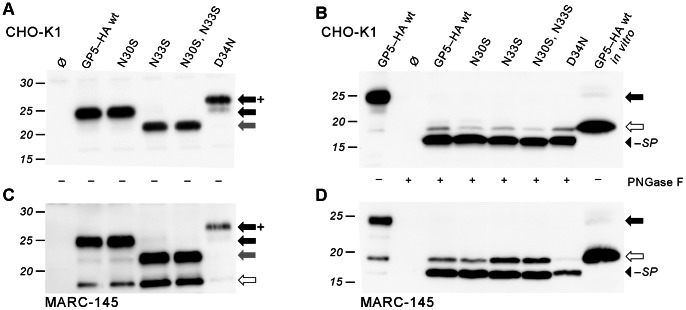
Processing analysis of cell expressed GP5–HA to reveal glycan-independent signal peptide cleavage. CHO-K1 (A, B) and MARC-145 (C, D; permissive for PRRSV) were transfected with plasmids encoding GP5–HA as wildtype (wt) and mutants with deleted or added glycosylation sites near the signal peptide cleavage site; Ø, empty plasmid control. Cell lysates were subjected to SDS-PAGE and Western blot (anti-HA tag) before (A, C) and after (B, D) deglycosylation with PNGase F. Molecular weight marker given on the left-hand side; arrows indicate sizes of unprocessed GP5–HA (white), glycosylated protein (black: wildtype glycosylation; grey: lacking one glycan; black/+: with one additional glycan), and deglycosylated protein without signal peptide (black arrowhead). In B and D, *in vitro*-generated GP5–HA (in the absence of microsomes, thus intrinsically unprocessed, i.e. not glycosylated and still containing the signal peptide, cf. Fig. 2) is shown in the rightmost lane for size comparison. Deglycosylated GP5–HA and all variants mostly ran faster than unprocessed protein.

**Figure 4:**
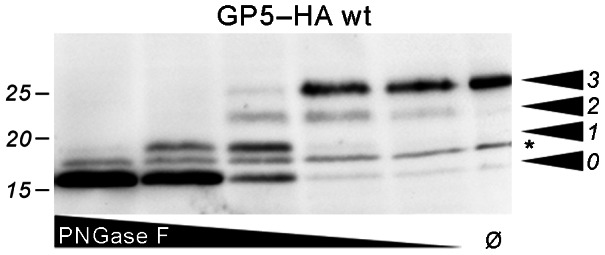
Limited PNGase F digestion of GP5–HA to show modification with three glycans. MARC-145 cells were transfected with the GP5–HA wt construct. Aliquots of the cell lysates were treated with decreasing concentrations of PNGase F as indicated or left undigested (Ø), then analyzed by SDS-PAGE and Western blot (anti-HA tag). The number of carbohydrates cleaved by PNGase F decreases with decreasing concentration. This causes a ladder-like appearance of bands that allows counting of the total number of carbohydrates linked to GP5–HA (arrowheads). The band denoted with the asterisk must not be counted since it is also present in untreated GP5–HA and probably represents unprocessed/non-translocated protein (see also Fig. 3C, white arrow).

We transfected CHO-K1 cells, which are known for good transfection efficiencies and expression rates, as well as MARC-145 cells, which are permissive for PRRSV and therefore particularly relevant for the assessment of GP5 processing [43]. SDS-PAGE and Western blot of cell lysates after transfection showed that all GP5–HA variants were expressed and apparently glycosylated (Fig. 3A, C). The comparison of electrophoretic mobilities between mutants shows that GP5–HA N30S ran at the same height as the corresponding wildtype. The size of the N33S as well as the N30S, N33S mutant appears to be reduced by roughly 2.5 kDa (one glycan). Limited digestion of GP5–HA wt with PNGase F digestion showed that GP5 comprised three glycans (Fig. 4). Thus, all the potential glycosylation sites except N30 (i.e., N33, N44, and N51) were indeed used, which is in line with previous investigations on PRRSV-GP5 [44]. The major band of GP5–HA D34N is increased in size by one additional glycan, showing that the additionally introduced glycosylation site is used. The (weaker) band at the height of wildtype protein indicates that this additional glycosylation is not realized in every molecule, probably because the glycosylation sequons of N33 (N^33^N^34^S^35^) and N34 (N^34^S^35^S^36^) overlap.

Expression and glycosylation of the GP5–HA constructs was comparable in CHO-K1 and MARC-145 cells. In the latter, however, there was a prominent fraction of protein running at the height of unprocessed protein as well (Fig. 3C). It is unclear whether such a protein (which is likely to remain in the cytosol) fulfills a specific function during the viral replication cycle or whether it is an artifact of the expression system.

To assess the influence of glycosylation on signal peptide cleavage, the lysates of the cells expressing variants of GP5–HA was subjected to PNGase F digestion prior to SDS-PAGE and Western blot. Representative results are shown in Fig. 3B (CHO-K1 cells) and Fig. 3D (MARC-145 cells). A prominent band at 16 kDa was observed in the case of GP5–HA wt as well as all the mutants under study. This protein clearly ran further in the gel than unprocessed GP5–HA obtained from *in vitro* transcription/translation in the absence of microsomes (thus not containing any glycans and still having the signal peptide, cf. Fig. 2), which was loaded on the gel as well as a control (rightmost lane in Fig. 3B/D). Yet, there is also a relatively weak band at the height of unprocessed protein, especially in the case of MARC-145 cells (Fig. 3D), which most likely corresponds to unprocessed rather than deglycosylated protein since such a band was also present in the lysate before PNGase F digestion (Fig. 3C).

To sum up, the signal peptide of GP5–HA appears to be cleaved efficiently, independently of whether there are glycans in the region of the signal peptide cleavage site. Furthermore, there were no discernible differences in migration between the different GP5–HA variants after deglycosylation with PNGase F, thus, the presence or absence of glycans did not markedly shift the signal peptide cleavage site.

### Signal Peptide Cleavage of GP5 in Various Porcine Cells

**Figure 5:**
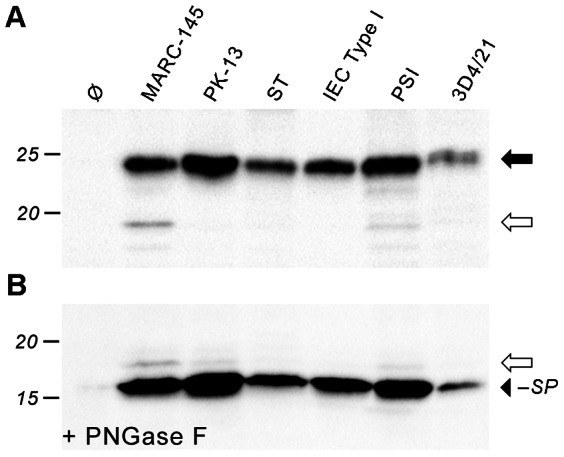
The signal peptide of GP5–HA is cleaved in different porcine cells. GP5–HA wt was expressed in MARC-145 cells as well as different porcine cells, which were lyzed and analyzed by SDS-PAGE and Western blot (anti-HA tag) before (A) and after (B) deglycosylation with PNGase F. Labels as in Fig. 3. GP5–HA is processed in the same manner in all cell types tested: PK-13 (porcine kidney), ST (testis), IEC Type I (intestine), PSI (small intestine), and 3D4/21 (alveolar monocytic cell line that can be rendered permissive to PRRSV by expression of the receptor CD163 [56]).

The “decoy epitope” hypothesis predicts that at later time points during natural infection of pigs, GP5 without “decoy epitope” is produced that causes the generation of neutralizing antibodies. Thus, it is possible that different porcine cells process GP5 differentially resulting in a protein with uncleaved signal peptide. To test signal peptide cleavage in porcine cells we first isolated porcine alveolar macrophages (PAMs) from the lavage of pig lung, but we were not able to detect expression of GP5–HA in these PAMs using a variety of transfection procedures (data not shown). Since primary cells are inherently difficult to transfect, we analyzed processing of GP5 in various porcine cell lines, i.e. monocytes and cells from intestine, kidney and testis. Cells were transfected with the GP5–HA wt construct; lysates were subjected to PNGase F digestion (Fig. 5B) or left untreated (Fig. 5A) and probed by SDS-PAGE and Western blot. For comparison of protein sizes, a sample from MARC-145 cells treated in the same manner was included in the analysis (see Fig. 3). All the samples from porcine cells had the same electrophoretic behavior as this control sample. Thus, no differences in the use of glycosylation sites were obvious, and the signal peptide was cleaved in all porcine cells tested in the same way as in MARC-145 cells. Hence, different porcine cell types do not differ with respect to processing of GP5.

### Signal Peptide Cleavage of GP5 from Various PRRSV Strains

**Figure 6:**
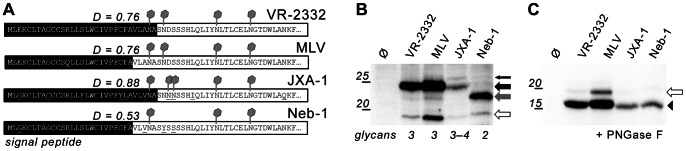
The signal peptide of GP5 from different PRRSV type 2 strains is cleaved. (A), N-terminal sequences (residues 1–60) of representative PRRSV type 2 strains (VR-2332: prototype strain, MLV: modified live vaccine “RespPRRS” (containing just one exchange – R13Q – in the signal peptide/ectodomain region relative to VR-2332), JXA-1: Chinese virulent strain, Neb-1: US-American virulent strain) with potential glycosylation sites (grey), predicted signal peptide (black), and propensity of signal peptide cleavage (D score according to SignalP 4.0, www.cbs.dtu.dk/services/SignalP/). Note that the difference in signal peptide cleavage at site 1 and 2 was small as shown in detail in Table S1. (B/C), MARC-145 cells were transfected with GP5–HA with the signal peptide/ectodomain sequence as depicted in (A), or with empty plasmid (Ø), subsequently lysed and assessed by SDS-PAGE and Western blot (anti-HA tag) before (B) and after (C) PNGase F digestion to remove glycans. Labels as in Fig. 2; the thin black arrow denotes additional glycosylation partially achieved in JXA-1; the number of glycans in the mature proteins is indicated on the bottom. The black arrowhead in (C) indicates the position of deglycosylated GP5–HA without signal peptide.

Next, it asked whether there are differences in signal peptide cleavage between GP5 proteins from different PRRSV type 2 strains. We chose the representative virulent strain JXA-1 from the outbreak in China [44] with a particularly high signal peptide cleavage probability for GP5 (D score: 0.88, SignalP 4.0), as well as GP5 from the US-American virulent strain Neb-1 [27], [45], for which a remarkably low signal peptide cleavage probability is calculated (D score: 0.53, just above the threshold level). In addition, GP5 from the attenuated modified live vaccine (MLV) strain RespPRRS, which is derived from VR-2332 [46], was also included in the analysis. While the various GP5 sequences all contain the conserved glycosylation sites N44 and N51, they differ with respect to the number of potential glycosylation sites in the hypervariable region: GP5 of VR-2332 and RespPRRS contain two potential glycosylation sites at positions 30 and 33 (of which only N33 appears to be used in VR-2332, see Figures 2 and 3), GP5 from JXA-1 has one additional site at position 34, and the sequence from Neb-1 just comprises one glycosylation site in the hypervariable region, located at position 30. Figure 6A depicts the signal peptide/ectodomain sequences of the GP5 variants under study, showing the number and position of potential glycosylation sites and the most probable cleavage site according to SignalP 4.0. However, only minor differences in the probability for cleavage at site 1 and site 2 were predicted as presented in Table S1.

These different GP5 constructs, each with *C*-terminal HA tag, were subjected to the same analysis as outlined above (transfection of MARC-145 cells, lysis, PNGase F digestion, SDS-PAGE and Western blot) to assess glycosylation and signal peptide cleavage (Fig. 6). GP5–HA from MLV had the same size as from VR-2332, indicating the same glycosylation pattern, i.e. no glycosylation at the site N30. GP5–HA from JXA-1 produced also a (minor) band with higher molecular weight, denoting that the overlapping sequons at position 34 and 35 (**NN**SS) are both glycosylated in a subfraction of molecules. Expression of GP5–HA from Neb-1 produced a band with a lower molecular weight, which implies that only the two conserved glycosylation sites, but not the glycosylation site at N30 are used. Note that GP5 variants of VR-2332 and RespPRRS produced a very weak band with a similar molecular weight. This indicates that one of the glycosylation sites is not used in every molecule (see also Fig. 1 and 2 for a similar band).

After deglycosylation of the different GP5–HA variants, all of them showed the same electrophoretic mobility (Fig. 6C). Thus, no difference in signal peptide processing could be discerned between GP5 variants from different strains. Note that also GP5–HA derived from Neb-1 was efficiently cleaved much though it is predicted to have a just-above-the-threshold probability.

### Analyzing Signal Peptide Cleavage of GP5 from Virus Particles and from a Recombinant GP5–M Dimer by Mass Spectrometry

**Figure 7:**
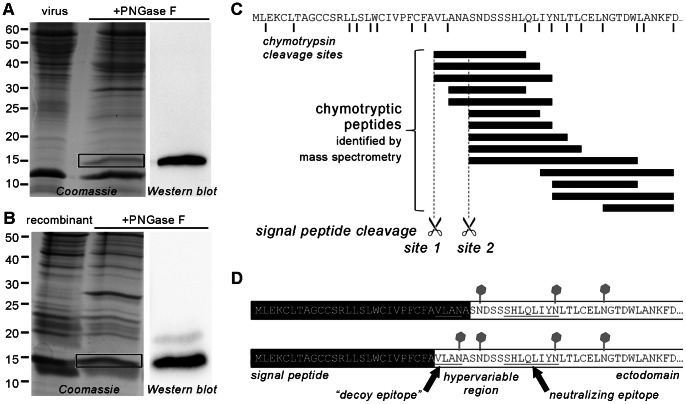
Identification of the signal peptide cleavage site of GP5 (virus-derived and recombinant) by mass spectrometry. (A), PRRSV (strain VR-2332) was grown in MARC-145 cells, precipitated with PEG-8000, pelleted and subjected to sucrose density gradient centrifugation. The virus-containing fraction was left untreated or deglycosylated with PNGase F and separated by reducing SDS-PAGE followed by Coomassie staining (left-hand side) or Western blot (anti-GP5 antiserum, right-hand side). The deglycosylated band corresponding to GP5 (black box) was cut out of the gel, digested with trypsin or chymotrypsin and analyzed by LC-MS/MS. (B), PRRSV GP5 (with His tag) and M (with HA tag) were co-expressed in *Sf*9 insect cells by infection with recombinant baculovirus. Following cell harvesting and lysis, GP5–M was enriched using Ni-NTA agarose (binding to GP5–His). The eluated protein was left untreated or digested with PNGase F and subjected to reducing SDS-PAGE and Coomassie staining (left-hand side) or Western blot (anti-His-tag antibody, right-hand side). The deglycosylated GP5 band (black box; coinciding with M) was cut out and treated as in (A). (C), representative result from mass spectrometry of virus-derived GP5 (as in A). The first 61 residues of GP5 are shown with the positions of the predicted chymotrypsin cleavage sites (black lines) and the putative signal peptide cleavage sites (broken lines). Chymotryptic peptides that were identified are represented as black bars. The pattern of peptides is evidence for signal peptide cleavage at sites 1 and 2. No peptides corresponding to the signal peptide region (1–26) were identified. (D), Conclusion from mass spectrometry, showing the *N*-terminal sequence of GP5 with signal peptide (black), glycosylations (grey) and the positions of the neutralizing and the “decoy epitope”. Two GP5 species exist with signal peptide cleavage at sites 2 (top) and 1 (bottom), respectively.

The observed migration pattern of deglycosylated GP5–HA is clear evidence for signal peptide cleavage; yet, the exact site of signal peptide cleavage cannot be derived from these data. We therefore employed mass spectrometry as a powerful tool to unequivocally identify proteins and protein fragments. Immunoprecipitation of cell-expressed GP5–HA does not yield enough material for mass spectrometry (data not shown). Therefore, we grew large quantities of PRRSV, strain VR-2332, in MARC-145 cells and partially purified the virus by PEG-8000 precipitation, ultracentrifugation and sucrose density gradient centrifugation (Fig. 7A). Virus proteins were subjected to PNGase F digestion to remove glycans and were separated by SDS-PAGE and stained with Coomassie. Deglycosylation is necessary to avoid heterogeneity in peptides due to the presence of differentially processed carbohydrate side chains. In addition, as a result of deglycosylation with PNGase F, asparagine is converted into aspartic acid and thus the occurrence of D instead of N is evidence that a certain glycosylation site is actually used. The band corresponding to GP5 (running at 16 kDa only after PNGase F digestion) was excised from the gel (see Fig. 7A). The band was verified to be GP5 by Western blot run in parallel, yielding a signal for GP5 at the same height as the excised band (Fig. 7A, right-hand panel). Two independent preparations were carried out.

In addition, GP5 (with a *C*-terminal His tag) was expressed in *Sf*9 insect cells by use of the baculovirus expression system together with the M protein, to which it is covalently linked by a disulfide bond (data not shown). The protein dimer was enriched using Ni-NTA, deglycosylated with PNGase F and separated by reducing SDS-PAGE to excise the band corresponding to GP5, the identity of which was confirmed by Western blot (anti-His tag, Fig. 7B). The proteins were digested with either trypsin or chymotrypsin, resulting peptides were eluted from the gel slices and subsequently subjected to liquid chromatography-tandem mass spectrometry (LC-MS/MS).

Processed GP5 was identified with high confidence in all samples as the protein was the first hit in the MASCOT search, where mass spectrometric fragments are matched with database entries to determine protein identity. In the samples subjected to trypsin digest, the fragment S**D**DSSSHLQLIYDLTLCELDGTDWLANK was detected. This corresponds to the residues 32–59 of GP5 and thus the *N*-terminal fragment of GP5 after signal peptide cleavage at site 2 (A31|S32). This peptide contains three aspartic acid residues (D) instead of the asparagine residues N33, N44 and N51, indicating that all three sites were used. However, the mass of the peptide with unmodified glycosylation site N33 (S**N**DSSSHLQLIYDLTLCELDGTDWLANK) was also present. This implies that not every GP5 molecule was glycosylated at N33. More importantly, no peptides corresponding to signal peptide cleavage at site 1 (A26|V27) or any other sites were recognized, nor were there any hints for GP5 fragments with the signal peptide retained at the *N*-terminus.

By chymotrypsin digest, more peptides were detected due to the existence of more cleavage sites. The identified peptides that correspond to parts of the GP5 ectodomain are listed in Table S2 and shown in Fig. 7C as black bars. Similarly to the trypsin digestion, there were peptides originating from GP5 with signal peptide cleavage at site 2 (A31|S32). Surprisingly though, fragments derived from GP5 starting with V27 were also detected, indicating that cleavage also occurred at site 1 (A26|V27). Regarding glycosylation, up to three varieties of a peptide were identified that differed in the modification of residues 30 and 33. For example, the peptide 27–40 was detected as VLADASDDSSSHLQ, VLANASDDSSSHLQ and VLANASNDSSSHLQ, containing a D at position 30 and 33, a D only at position 33 or no D. This indicates that N30 located between cleavage sites 1 and 2 was glycosylated in a subfraction of GP5 molecules, but only if N33 was also glycosylated.

Peptides starting with V27 as well as with S32, but not those containing sequences from more *N*-terminal parts of the molecule, were identified also in GP5 expressed with the baculovirus system. This is evidence for use of signal peptide cleavage sites 1 (A26|V27) and 2 (A31|S32). Likewise, the sequence of those peptides also indicates that either both N30 and N33, or only N33 or none of those sites were glycosylated.

Taken together, this experimental outcome implies that GP5’s signal peptide is cleaved from every GP5 molecule. Surprisingly, two cleavage sites, i. e. between A26 and V27 and between A31 and S32 were identified. These are the two sites that yielded values above the threshold in signal peptide cleavage predictions with SignalP 4.0. These two variants of GP5 are displayed in Fig. 7D. Unfortunately, due to the number of peptides, quantitative estimation is not feasible, but there was a tendency towards a higher proportion of peptides derived from signal peptide cleavage site 2. Thus, the sequence of the “decoy epitope” – situated between site 1 and site 2– is present in a subset of mature GP5. A subpopulation of GP5 molecules with the decoy epitope sequence contains a carbohydrate at position 30, i.e. within the proposed antibody binding region.

### The Major Fraction of GP5 in Transfected Cells is Cleaved at Signal Peptide Cleavage Site 2

Since the relative abundance of the two GP5 species could not be determined by mass spectrometry, we endeavored to discriminate biochemically between the two species. To this end, we engineered artificial mutants of GP5–HA where signal peptide cleavage is blocked by replacing relevant small, non-polar residues near the cleavage sites by bulkier amino acids. SignalP 4.0 was employed to evaluate and optimize these substitutions; the results are given in Table S1. To generate a variant of GP5–HA with uncleavable signal peptide (GP5–HA uncl.), the alanines at positions 26, 29 and 31 were replaced by phenylalanine, tyrosine and phenylalanine, respectively. To obtain GP5–HA cleaved at exclusively site 1 (GP5–HA cl.1), site 2 was blocked by the mutations A29S, A31Y. Likewise, cleavage at site 1 was blocked by the mutations A26F, A29S (cleavage expected to be at site 2 only, mutant GP5–HA cl.2).

**Figure 8:**
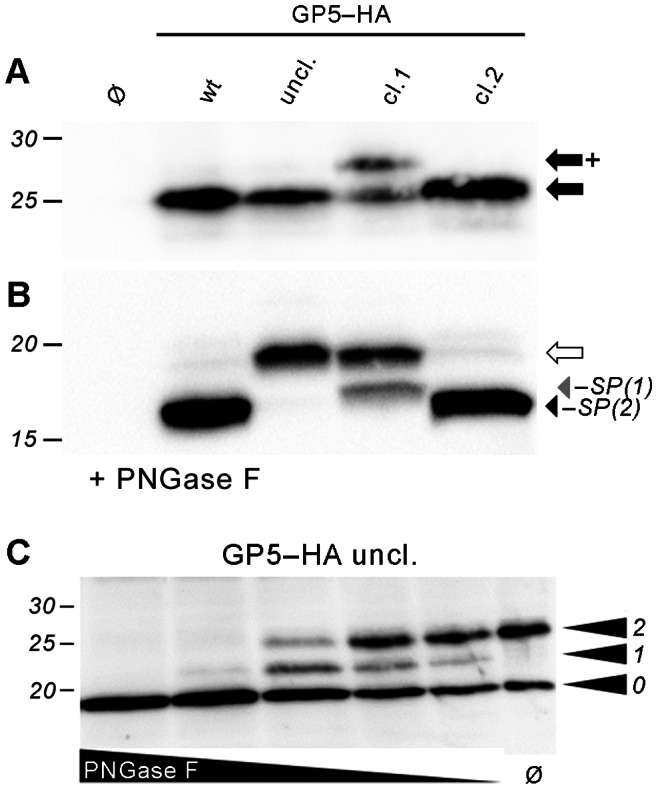
The majority of GP5–HA molecules in transfected cells is cleaved at site 2. MARC-145 cells were transfected with variants of GP5–HA or with empty plasmid (Ø), subsequently lysed and assessed by SDS-PAGE and Western blot (anti-HA tag) before (A) and after (B) PNGase F digestion to remove glycans. wt: GP5–HA wildtype; uncl.: Signal peptide (SP) cleavage completely blocked by mutation A26F, A29Y, A31F; cl.1: SP cleavage possible at site 1 (A26|V27) only (mutation A29S, A31Y); cl.2: SP cleavage at site 2 (A31|S32) only (mutation A26F, A29S). Black arrow: GP5–HA with cleaved SP, carrying three glycans, and/or GP5–HA comprising SP and two glycans; black arrow with plus sign: GP5–HA, SP cleaved, four glycans; white arrow: GP5–HA unprocessed/deglycosylated and containing the SP; grey and black arrowhead: GP5–HA with SP cleavage at site 1 or 2, respectively. (C), limited PNGase F digestion of GP5–HA uncl., performed and labeled as in Fig. 4. The protein carries two glycans.

Wildtype GP5–HA and these mutants were expressed in MARC-145 cells and subjected to Western blot analysis before and after PNGase F digestion of lysates (Fig. 8). For GP5–HA uncl., a single band at the position of wildtype GP5–HA was detected before digestion with PNGase F (Fig. 8A, third lane), although a size increase by the retained signal peptide would be expected. However, after removal of glycans by PNGase F (Fig. 8B), this mutant exhibited a retarded SDS-PAGE mobility relative to GP5–HA wt and ran at the position expected for protein with uncleaved signal peptide (19 kDa). Limited PNGase F digestion revealed that GP5–HA uncl. carries only two glycans (Fig. 8C). It can be assumed that the glycosylation sites N30 and N33, albeit present in the protein, were not modified, most probably due to being held too close to the membrane by the uncleaved signal peptide that might function as a signal-anchor domain in that case.

GP5–HA cl.1 exhibited two bands after digestion with PNGase F. The major band corresponds in size to GP5–HA uncl., implying that the mutations unexpectedly prevented cleavage of the whole signal peptide in a large fraction of molecules. The minor band of deglycosylated GP5–HA cl.1 ran clearly lower than GP5–HA uncl., but higher compared to GP5–HA wt and GP5–HA cleavable at site 2 only, indicating that it was cleaved at site 1 (Fig. 8B). Two bands were also present for GP5–HA cl.1 before digestion with PNGase F – a major band at the height of GP5–HA wt/uncl./cl.2, and a weaker band increased in size by about 3 kDa (Fig. 8A). The latter most likely corresponds to GP5–HA with signal peptide cleavage at site 1 and carrying an additional glycan at the site N30.

Most inportantly, before and after digestion with PNGase F, GP5–HA cl.2 ran exactly like wildtype, demonstrating that cleavage of the latter also occurs at site 2.

In conclusion, the difference in the SDS-PAGE mobility observed between GP5–HA cl.1 and cl.2 allows for discrimination between GP5–HA cleavage at signal peptide cleavage sites 1 and 2. As wildtype GP5–HA behaved exactly like GP5–HA cl.2, but unlike GP5–HA cl.1, it can be concluded that the vast majority of GP5–HA wildtype is cleaved at signal peptide cleavage site 2.

## Discussion

Despite the high medical and economic impact of PRRSV, molecular details of its structural proteins are still sparse, but relevant for virus pathogenicity. The major glycoprotein GP5 has considerable immunological relevance since the ectodomain harbors an epitope for neutralizing antibodies. However, the generation of neutralizing antibodies is retarded in PRRSV infection, which has been suggested to be one major cause for the persistent phenotype of PRRSV infections that makes the disease so difficult to control. GP5 from North American (type 2) strains were hypothesized to possess a non-neutralizing, but immunodominant “decoy epitope”, located upstream from the neutralizing epitope [32]. However, this “decoy epitope” is located in (or near) the signal peptide and therefore its presence in mature GP5 depends on whether (and where) the signal peptide is cleaved. Moreover, the use of potential glycosylation sites in the hypervariable region located between both epitopes could influence signal peptide cleavage.

**Figure 9:**
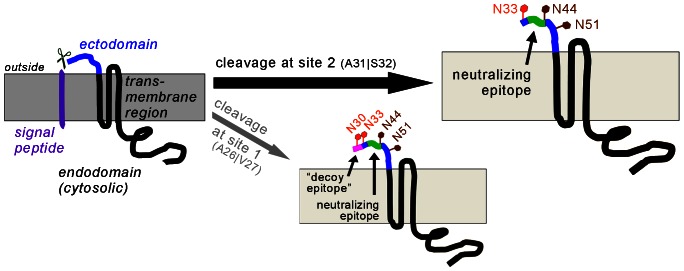
Conclusion – Major fraction of GP5 from PRRSV type 2 does not contain the “decoy epitope”. Schematic representation of GP5 fractions as evidenced in this study. The signal peptide of GP5 is predominantly cleaved at site 2 (A26|V27; black arrow), but is also cleaved in minor quantities at site 1 (A26|V27, thin grey arrow). The “decoy epitope” (magenta) is preserved only in GP5 cleaved at site 1. The neutralizing epitope (green) is present in either case. Heterogeneity occurs also at non-conserved glycosylation sites (red). The fraction of GP5 with the “decoy epitope” contains carbohydrates at either both N30 and N33, or only at N33 or none of these sites. A subfraction of site 2-cleaved GP5 does not contain a carbohydrate at N33. Conserved glycosylation sites are in brown.

Here we show that the signal peptide is cleaved from GP5 of the American PRRSV type 2 reference strain VR-2332, both upon *in vitro* translation in the presence of microsomes (Fig. 2) as well as in transfected cells (Fig. 3). Processing was not affected by deletion and insertion of glycosylation sites in the vicinity of the cleavage site, which also occurs in natural virus strains (Fig. 2+3). Likewise, the signal peptide was also cleaved from different GP5 variants derived from natural virus strains (attenuated and virulent ones). These differ regarding the amino acids in the vicinity of the signal peptide cleavage site and the number of glycans (Fig. 6). In addition, a variety of porcine cell lines processed GP5 in the same manner, as far as discernible by SDS-PAGE (Fig. 5). Mass spectrometry of peptides derived from the GP5 of virus particles and from a recombinant GP5/M dimer expressed in insect cells unequivocally demonstrated that the signal peptide is cleaved from every GP5 molecule (Fig. 7). Intriguingly, two different cleavage sites were identified, which are identical to the sites predicted by SignalP 4.0. Thus, there are probably two fractions of GP5 molecules present in virus particles, one with and one without the “decoy epitope”. By comparing the wildtype GP5 probe to mutants where either signal peptide cleavage site 1 or 2 was blocked, we obtained circumstantial evidence that the largest part of wildtype GP5 is cleaved at site 2 and will thus not contain the “decoy epitope” (Fig. 8). This is schematically displayed in Fig. 9.

Microheterogeneity was observed regarding the use of potential glycosylation sites in the hypervariable region. GP5 without decoy epitope was found to be present in virus preparations in two variants: one containing and the other lacking carbohydrates at position N33. The GP5 fraction containing the “decoy epitope” was even identified in three glycoforms: either without carbohydrates in the hypervariable region, glycosylated at N33 only or at both N30 and N33.

Since mass spectrometry is an inherently non-quantitative method, no definitive conclusions regarding the abundance of each GP5 species are possible. However, the biochemical experiments allow us to draw some conclusions about the use of glycosylation sites in the hypervariable region. Since exchange of N30 did not reduce the molecular weight of the GP5 probe (Fig. 2+3), this glycosylation site is apparently used only in a very small proportion of all GP5 molecules. In addition, limited digestion of GP5–HA wt revealed that GP5 contains three carbohydrates; a band with a higher molecular weight (implying four glycans) was never observed, even after prolonged exposure of the blots. In contrast, a weak GP5 band with only two carbohydrates was seen in several blots (see Fig. 2, 3, 6) suggesting that N33 is not glycosylated in every molecule. In summary, the vast majority of GP5 molecules is glycosylated at N33 and at the conserved glycosylation sites N44 and N51, confirming previous data [44].

The analysis of glycosylation in the hypervariable region also provides interesting insights into the cotranslational processing of GP5. Generally, efficient *N*-glycosylation occurs only if the glycosylation site is located at a certain distance (at least 12–14 amino acids) from the luminal membrane surface of the ER [47]. This condition is hardly fulfilled for the glycosylation sites N30 and N33 when the nascent protein is membrane-anchored by the signal peptide (see Fig. 1A). Accordingly, the GP5 variant with blocked signal peptide cleavage sites (GP5–HA uncl.) carried only two glycans (Fig. 8C). In addition, there was a detectable quantity of protein carrying four glycans upon analysis of the artificial mutant GP5–HA cl.1, where signal peptide cleavage is only possible at site 1 and N30 is thus preserved in the mature protein (Fig. 8A).Thus, it is plausible that signal peptide cleavage precedes glycosylation: First, signal peptide cleavage releases the protein stretch containing N33 (and N30, if cleavage is at site 1) from the membrane. This then allows the oligosaccharyl transferase to attach core glycosylation to these residues. If signal peptide cleavage and glycosylation of neighboring sites occur in such a sequential manner, it is unlikely that glycan addition has an impact on signal peptide cleavage. Indeed, deletion or addition of glycosylation sites did not impede signal peptide cleavage in any of our experiments.

While mass spectrometry could prove that there are mature GP5 molecules that contain the “decoy epitope” sequence, the combined biochemical data imply that the relative abundance of this GP5 species is small or even minute. Assuming that the GP5 variants are incorporated into virus particles at the same ratio as they are produced in transfected cells, mature virus particles presumably comprise only very few “decoy epitope”-containing GP5 molecules. In light of these data, it is hard to grasp how antibodies directed against such a small fraction of GP5 (cleaved at site 1) should mask access of antibodies against the putative neutralizing epitope. Besides, we cannot exclude that the fraction of GP5 molecules containing a “decoy epitope” identified in our virus preparations are actually a contamination, such as virus-like particles (containing GP5/M dimers enwrapped in a lipid membrane) that were not separated by gradient centrifugation from infectious particles.

Overall, our results challenge, but do not ultimately falsify the “decoy epitope” hypothesis, since the small fraction of “decoy epitope”-containing GP5 could still be significant. Ultimate clarification of this issue would require experimental infection of pigs with recombinant viruses harboring a homogenous population of GP5 molecules, either cleaved exclusively at site 1 (thus completely maintaining the “decoy epitope”) or exclusively at site 2 such that the “decoy epitope” is removed from all GP5 molecules. Planning of such mutations must also take into account the presence of the overlapping open reading frame 5a, which encodes the recently discovered membrane protein 5a [9], [10]. This was not considered when engineering the mutants GP5–HA cl.1 and cl.2. At least GP5–HA cl.2 was functionally processed in a uniform manner (Fig. 8), demonstrating that this approach could be feasible. Promising mutations should then be introduced into the viral genome to test whether recombinant PRRSV can be generated and whether it has growth properties similar to wildtype virus. Finally, experimental infection of pigs with these PRRSV variants could provide evidence whether the “decoy epitope” in GP5 is required for the induction of persistent infection. Especially recombinant viruses with GP5 completely lacking the “decoy epitope” might induce neutralizing antibodies more quickly and more robustly and could hence be promising candidates for a vaccine. The immunogenicity of such variants might be further improved by deletion of glycosylation sites in the hypervariable region that do not affect signal peptide cleavage *per se* (Figs. 2–4) and are not essential for virus replication, but induce higher levels of neutralizing antibodies [44], [48], [49].

## Material and Methods

### Ethics Statement

Animal experiments were approved by the local state office of occupational health and technical safety “Landesamt für Gesundheit und Soziales Berlin” (LaGeSo Reg. Nr. 0347/09).

### Cells

Cell lines CHO-K1 (Chinese hamster ovary cells, ATCC CCL-61), MARC-145 (simian kidney epithelial cells derived from MA-104 [43], ATCC CRL-11171), PK-13 (porcine kidney epithelial cells, ATCC CRL-6489), ST (swine testis cells, ATCC CRL-1746), IEC Type I (swine intestinal epithelial cells, [50]), PSI (porcine small intestinal cells, BioNutriTech, Lunel, France), and 3D4/21 (porcine alveolar monocytic cells, ATCC CRL-2843) were maintained in adherent culture in Dulbecco’s Modified Eagle’s Medium (DMEM, PAN, Aidenbach, Germany) supplemented with 10% fetal calf serum (Perbio, Bonn, Germany) at 37°C in an atmosphere with 5% CO_2_ and 95% humidity. Suspension-adapted *Sf*9 and TriEx *Sf*9 cells were cultured in Falcon Erlenmeyer flasks (BD Bioscience, Heidelberg, Germany) in serum-free medium SF-900 II SFM (Invitrogen, Karlsruhe, Germany) at 27°C with orbital shaking at 120 rpm.

### Plasmids

The nucleotide sequence encoding GP5 of PRRSV, strain VR-2332, was *in vitro*-synthesized (Eurofins MWG Operon, Ebersberg, Germany) including silent mutations to generate additional restriction sites. Additionally, the sequence encoding a *C*-terminal HA tag (amino acids YPYDVPDYA) was incorporated. The GP5-ORF was subcloned into the plasmid pCMV-TnT (Promega, Mannheim, Germany, containing T7 and CMV promoters) using *Xho*I and *Not*I restriction sites to yield pCMV-TnT–GP5–HA wt. Using this plasmid as a template, site-directed mutagenesis was performed by overlap extension polymerase chain reaction (PCR) using standard molecular biology techniques [51] to generate GP5–HA mutants (VR-2332 GP5 with mutations N30S; N33S; N30S, N33S; and D34N; other strains: mutagenesis in the signal peptide/ectodomain region: R13Q [Neb-1]; E3G, G9C, S16F, C24Y, F25L, A29V, D34N, S35N, L39I, N58Q [JXA-1] and E3G, A29V, N33Y, D34S [Neb-1]). All plasmids were amplified in *E. coli* XL-1 blue (Stratagene/Agilent, Waldbronn, Germany), purified (PureYield Maxi Prep System, Promega, Mannheim, Germany) and sequenced (GATC, Konstanz, Germany) before use in experiments.

For recombinant protein expression of the GP5–M complex using the baculovirus expression system, a bacmid was generated that comprises the recombinant baculovirus genome. First, the ORFs of GP5 and M were inserted into suitable MultiBac transfer plasmids [52], which were obtained from ATG:Biosynthetics (Merzhausen, Germany). The GP5 ORF was amplified from pCMV-TnT–GP5–HA by PCR for cloning into the pACEBAC1 acceptor plasmid. The nucleotide sequence encoding a *C*-terminal 7×histidine tag was incorporated into the reverse primer. Likewise, the ORF encoding the M protein was amplified from the pVR-V7 vector (full-length cDNA of PRRSV VR-2332, [53]) for cloning into the pIDC donor vector, including a *C*-terminal HA tag. Both plasmids (pACEBAC1–GP5–His and pIDC–M–HA) contain loxP sites to allow for plasmid fusion by Cre–lox recombination, which was performed *in vitro* using Cre recombinase (New England BioLabs, Frankfurt am Main, Germany) according to the supplier’s protocol, followed by transformation of *E. coli* DH5α and selection on Luria–Bertani broth agar plates with gentamycin and chloramphenicol. Plasmid DNA was checked for correctness by sequencing (GATC, Konstanz, Germany) and inserted into baculovirus bacmid DNA by recombination using the Bac-to-Bac system (Invitrogen, Karlsruhe, Germany).

### *In vitro* Transcription/Translation

GP5–HA was generated by *in vitro* transcription/translation using the TnT Quick Coupled Transcription/Translation System (Promega, Mannheim, Germany). For the synthesis of unprocessed protein, a reaction (25 µL) was typically composed of 20 µL rabbit reticulocyte lysate (TnT master mix, also including T7 RNA polymerase), 2.2 µL EasyTag Express [^35^S] protein labeling mix (radioactively labeled methionine/cysteine, 20 µCi, Perkin-Elmer), and 1 µg of pCMV-TnT–GP5–HA plasmid DNA. To synthesize processed protein, canine pancreatic microsomal membranes (Promega) or porcine pancreatic membranes (prepared as described below) were included in the reaction (typically 1.6 µL). Reactions were incubated for 90 min at 30°C. Subsequently, the products were supplemented with glycoprotein denaturing buffer (final concentrations: 0.5% SDS, 40 mM DTT) and incubated at 100°C for 10 min. For deglycosylation, aliquots of these denatured samples were digested with 50–100 units peptide-*N*-glycosidase (PNGase) F according to the manufacturer’s instructions (New England BioLabs, Frankfurt am Main, Germany) for 4 h at 37°C. Control samples were left untreated. The samples were supplemented with reducing SDS-PAGE loading buffer and assessed by SDS-PAGE and Western blot (see below). The employment of radioactively labeled amino acids allowed detection of *in vitro*-synthesized protein by fluorography (as described, [54]), albeit with numerous unspecific bands. Expression levels of individual constructs varied largely from experiment to experiment (results not shown).

### Preparation of Porcine Pancreas Microsomes

Microsomal membranes were isolated from 20 g of pancreas tissue from slaughtered pigs by a protocol adapted from Walter & Blobel [55]. After mechanical removal of connective tissue, the pancreas, kept in ice-cold buffer A (250 mM sucrose, 50 mM triethanolamine, 50 mM potassium acetate, 6 mM magnesium acetate, 1 mM EDTA, 1 mM DTT, 0.5 mM PMSF, pH 7.5, with Complete protease inhibitor cocktail [Roche]; 4 mL/g tissue), was homogenized with a fruit shredder followed by 42 strokes with a Dounce homogenizer. Debris was pelleted by low-speed centrifugation (600× g, 10 min, 4°C), the supernatant was then subjected to a series of centrifugation steps to remove larger organelles (twice at 10,000 rpm in a Beckman Ti-45 rotor for 20 min, and then through a 1.3 M sucrose cushion in buffer A at 32,000 rpm in a Ti-45 rotor for 150 min). The resulting white pellet was resuspended in buffer B (250 mM sucrose, 50 mM triethanolamine, 1 mM DTT, pH 7.5 adjusted with acetic acid, 1.5 mL), supplemented with EDTA (25 mM), incubated on ice for 30 min and then pelleted through a 1.3 M sucrose cushion in a Beckman Sw-55-Ti rotor at 40,000 rpm for 75 min, resuspended in buffer B (1.5 mL), aliquotted, flash-frozen in liquid nitrogen and stored at −80°C.

### Protein Processing Analysis in Cells

To assess processing of GP5–HA in cells, cells were seeded in 35-mm dishes and transfected with 4 µg of pCMV-TnT–GP5–HA plasmid DNA using TurboFect (Fermentas/Thermo, St. Leon-Rot, Germany). 24 h after transfection, cells were washed with phosphate-buffered saline (PBS), detached from the dish with trypsin–EDTA (PAN Biotech, Aidenbach, Germany), pelleted, resuspended in 80 µL glycoprotein denaturing buffer (0.5% SDS, 40 mM DTT) and boiled for 10 min at 100°C. Typically, 15 µL of this lysate was digested with PNGase F (2.5–5 units/µL, 4 h at 37°C) according to the manufacturer’s instructions (New England BioLabs, Frankfurt am Main, Germany). For limited PNGase F digestion, a serial twofold dilution of PNGase F (starting with 0.6 units/µL) was prepared for incubation with aliquots of the lysate in the same manner. Control reactions were left untreated. After the deglycosylation reaction, samples were supplemented with reducing SDS-PAGE loading buffer and subjected to SDS-PAGE and Western blot.

### SDS-PAGE and Western Blot

After sodium dodecyl sulfate-polyacrylamide gel electrophoresis (SDS-PAGE), typically using 15% polyacrylamide gels, gels were either stained with Coomassie Brilliant Blue G-250 (Sigma-Aldrich, Taufkirchen, Germany) or blotted onto polyvinylenedifluoride (PVDF) membrane (GE Healthcare, Freiburg im Breisgau, Germany) using standard methodology. After blocking of membranes (blocking solution: 5% skim milk powder in PBS with 0.1% Tween-20) for 1 h at 25°C, antibody was applied for 16 h at 4°C in blocking solution: rabbit-anti-HA tag antibody (ab9110, Abcam, Cambridge, UK, 1∶4,000) was used to detect HA-tagged GP5, mouse-anti-His tag antibody (H1029, Sigma-Aldrich, Taufkirchen, Germany, 1∶3,000) was employed for recombinant GP5–His, and virus-derived GP5 was detected with a polyclonal rabbit antiserum raised against peptide LDTKGRLYRWRSPC, which corresponds to residues 146–158 of PRRSV VR-2332 GP5 with *C*-terminal cysteine (Genosphere Biotechnologies, Paris, France, 1∶1,000). After washing (3× 10 min with PBST), suitable horseradish peroxidase-coupled secondary antibody (anti-rabbit or anti-mouse, Sigma-Aldrich, Taufkirchen, Germany, 1∶5,000) was applied for 45 min at 25°C. After washing, signals were detected by chemiluminescence using the ECLplus reagent (Pierce/Thermo, Bonn, Germany) and a Fusion SL camera system (Peqlab, Erlangen, Germany).

### Preparation of PRRSV

MARC-145 cells in ten 15-cm dishes were infected with PRRSV, strain VR-2332, at a multiplicity of infection (MOI) of 0.01 for 2 h and incubated for 4 days at 37°C, 5% CO_2_ in DMEM +5% FCS. Upon occurrence of cytopathic effect, the supernatant was harvested, cleared by low-speed centrifugation (3,000× g, 5 min) and supplemented with polyethylene glycol (PEG)-8000 (Sigma-Aldrich, Taufkirchen, Germany) to a final concentration of 10% (w/v) at 4°C with gentle shaking for 18 h. The PEG-8000 precipitate was pelleted at 17,700× g for 1 h at 4°C (Beckman JLA-16,250), resuspended in HNE buffer (50 mM HEPES, 100 mM NaCl, 1 mM EDTA, pH 7,5) and applied on top of a sucrose density gradient (20–60% (w/v) in TNE buffer: 10 mM Tris·HCl, 10 mM NaCl, 1 mM EDTA, pH 7.5), which was ultracentrifuged for 18 h at 35,000 rpm, 4°C, in a Beckman SW-40 rotor. No distinct virus band was visible. The gradient was divided into fractions, which were pelleted (Beckman SW-28, 27,000 rpm, 2 h, 4°C) and resuspended in TNE buffer. After testing of the fractions for the presence of GP5 by Western blot, the virus-containing fraction was denatured with glycoprotein denaturing buffer (0.5% SDS, 40 mM DTT) for 10 min at 100°C and then deglycosylated with PNGase F (New England BioLabs, Frankfurt am Main, Germany) for 4 h at 37°C. Proteins were then separated by SDS-PAGE. The band corresponding to GP5 was excised with a scalpel and processed for mass spectrometry (see below).

### Production of Recombinant Baculovirus, Expression and Enrichment of GP5–M

*Sf*9 insect cells (8×10^5^ cells) were seeded in a six-well plate in Grace’s insect medium and allowed to attach (15 min at room temperature), then transfected with 2 µg of bacmid DNA using Cellfectin II (Invitrogen, Karlsruhe, Germany) according to the supplier’s protocol. Four hours after transfection, medium was replaced by Sf-900 II medium and cells were incubated for further 72 h at 27°C. Subsequently, supernatants were harvested, cleared (centrifugation at 1,000× g for 5 min), titrated (baculoQUANT all-in-one kit, Oxford Expression technologies, Oxford, UK) and stored at 4°C. Amplification of this “P1 virus stock” was performed by infection of 50 mL TriEx *Sf*9 cells with this supernatant at an MOI of 0.1 and incubation in suspension culture (130 rpm) for 72 h, followed by collecting the supernatant and titration. For protein expression, 1×10^9^ TriEx *Sf*9 cells at a density of 2×10^6^ cells/mL were infected with high-titer virus (MOI 2) and incubated in suspension culture. 72 h later, cells were pelleted (4,000× g, 10 min), washed twice with PBS and stored at −80°C before lysis.

For enrichment of the GP5–M complex, cells were lysed in 80 mL of lysis buffer (50 mM NaPO_4_, pH 8.0, 500 mM NaCl, 1% Triton X-100, 10 mM imidazole and Roche Complete protease inhibitors) on ice for 45 min, then cleared from cellular debris by centrifugation at 40,000× g for 45 min. Two milliliters of Ni-NTA agarose (Qiagen, Hilden, Germany) was equilibrated by washing twice with lysis buffer. Washed Ni-NTA agarose beads were added to the clarified cell lysate and incubated for one hour with occasional shaking at 4°C to allow binding of His-tagged GP5. Beads were washed twice with lysis buffer, five times with 2 mL wash buffer 1 (50 mM NaPO_4_, pH 8.0, 500 mM NaCl, 0.2% Triton X-100, 20 mM imidazole), five times with 2 mL wash buffer 2 (50 mM NaPO_4_, pH 8.0, 500 mM NaCl, 0.2% Triton X-100, 40 mM imidazole). The protein was then eluted in five 1-mL fractions using the elution buffer (50 mM NaPO_4_ pH 8.0, 500 mM NaCl, 0.2% Triton X-100, 250 mM imidazole). The protein was then deglycosylated using PNGase F (New England BioLabs, Frankfurt am Main, Germany) and separated by SDS-PAGE, the band corresponding to GP5 was excised with a scalpel.

### Protein Sample Preparation for Mass Spectrometry

After SDS-PAGE separation of proteins, excised protein bands were washed with 50% (v/v) acetonitrile in 50 mM ammonium bicarbonate/(NH_4_)HCO_3_, shrunk by dehydration in acetonitrile, and dried in a vacuum centrifuge. Disulfide bonds were reduced by incubation in 60 µL of 10 mM DTT in 50 mM (NH_4_)HCO_3_ for 45 min at 56°C. Alkylation was performed by replacing the DTT solution with 55 mM iodoacetamide in 50 mM (NH_4_)HCO_3_. The gel pieces were shrunk by dehydration in acetonitrile, dried in a vacuum centrifuge, re-swollen in 20 µL of 50 mM (NH_4_)HCO_3_containing 100 ng trypsin (Promega, Madison, WI, USA), and incubated at 37°C overnight. In the case of chymotrypsin, excised protein bands were incubated with 110 ng of enzyme (Roche Diagnostics, Penzberg, Germany) in 20 µL of 50 mM (NH_4_)HCO_3_ for 20 h at 25°C. Peptides were extracted using 20 µL of 0.5% (v/v) trifluoroacetic acid (TFA) in acetonitrile and the separated liquid was dried under vacuum. The samples were reconstituted in 6 µL of 0.1% (v/v) TFA and 5% (v/v) acetonitrile in water.

### Liquid Chromatography-tandem Mass Spectrometry

LC-MS/MS analyses were performed on an Orbitrap Elite hybrid mass spectrometer (Thermo Fisher, Bremen, Germany) equipped with an UltiMate 3000 LC (Dionex). LC separations were performed on a capillary column (Acclaim PepMap100, C18, 2 µm, 100 Å, 150 mm×75 µm i.d., Dionex) at an eluent flow rate of 200 nL/min using a linear gradient of 3–30% eluent B in 33 min with further increase to 80% B at 40 min. Mobile phase A contained 0.1% (v/v) formic acid in water, mobile phase B contained 0.1% (v/v) formic acid in acetonitrile. MS data were acquired in a data-dependent strategy selecting MS/MS fragmentation events based on the precursor abundance in the MS scan. LTQ MS/MS spectra were acquired with a target value of 20,000 ions. The maximum injection time for MS/MS was 300 ms, the dynamic exclusion time was 30 s.

### Data Processing

MS and MS/MS spectra were used to search against a custom-made database containing all proteins of the SwissProt 2010_7 database (521,024 sequences; 183,901,752 residues) including the full-length GP5 sequence. In addition, the database contains all possible *N*-terminally truncated sequences of GP5 resulting from signal peptide cleavage site prediction in the range between residue 20 and 40. Asn/Asp amino acid exchanges were used as variable modifications.For identification of GP5 peptides, the processed MS/MS spectra were compared with the theoretical fragment ions of GP5 peptides using the MASCOT server version 2.2.2 (Matrix Science Ltd., London, UK). The maximum of two and six missed cleavages was allowed for tryptic and chymotryptic peptides, respectively. The mass tolerance of precursor and sequence ions was set to 10 ppm and 0.35 Da, respectively.

## Supporting Information

Table S1 **Amino acid sequences and signal peptide cleavage site prediction for the GP5 sequences analyzed in this study.** For all GP5 variants under study, the sequence of residues 20–40 is listed along with parameters from signal peptide cleavage prediction using SignalP 4.0 (www.cbs.dtu.dk/services/SignalP/): D score (likelihood of signal peptide cleavage immediately before the site given in brackets), and Y scores for “site 1” (A26|V27) and “site 2” (A31|V32). The higher Y, the more likely signal peptide cleavage at this site. Values above 0.5 can be considered “above the threshold”. Most probable cleavage site is indicated by Y score in **bold**. – In the sequence, the residues in the predicted signal peptide are in small letters, the residues in the mature protein in capital letters. Potential glycosylation sites according to the sequon **N**XS/T are annotated in bold, the “decoy epitope” sequence (*VLAN*) is in italics in the GP5 sequence from VR-2332 wt. – GenBank references: VR-2332 [AAD12129.1], MLV RespPRRS [AAD27656.1], JXA-1 [ABL60902.1], Neb-1 [ACE87854.1]. Full-length protein sequences were submitted to the SignalP 4.0 prediction.
(DOCX)

Table S2 **Chymotryptic peptides identified by mass spectrometry from virus-derived GP5 and from recombinantly expressed GP5.** Representative list of ectodomain peptides of deglycosylated GP5, digested with chymotrypsin, that were identified by mass spectrometry (LC-MS/MS), source: enriched PRRS virus particles and recombinant protein expression of GP5–M in *Sf*9 insect cells (Baculovirus system). Sequences of peptides along with their observed and calculated masses, number of missed chymotrypsin cleavage sites, and their ions score (as a measure of confidence of the mass match and taken from the MASCOT program). Note that glycosylated asparagine (N) is converted to aspartic acid (D) by deglycosylation with PNGase F. Peptides starting with V27 as well as with S32 were identified in both types of sample, which is evidence for use of signal peptide cleavage sites 1 (A26|V27) and 2 (A31|S32). – * The sequence between the dots indicates the peptide which was identified by MS/MS.
(DOCX)

## References

[1] Snijder EJ, Meulenberg JJ (1998) The molecular biology of arteriviruses. J Gen Virol 79: 961–979. 9603311 10.1099/0022-1317-79-5-961

[2] Wensvoort G, Terpstra C, Pol JM, ter Laak EA, Bloemraad M, et al (1991) Mystery swine disease in The Netherlands: the isolation of Lelystad virus. Vet Q 13: 121–130. 1835211 10.1080/01652176.1991.9694296

[3] Collins JE, Benfield DA, Christianson WT, Harris L, Hennings JC, et al (1992) Isolation of swine infertility and respiratory syndrome virus (isolate ATCC VR-2332) in North America and experimental reproduction of the disease in gnotobiotic pigs. J Vet Diagn Invest 4: 117–126. 1616975 10.1177/104063879200400201

[4] Zhou L, Yang H (2010) Porcine reproductive and respiratory syndrome in China. Virus Res 154: 31–37. 20659506 10.1016/j.virusres.2010.07.016

[5] Tian D, Wei Z, Zevenhoven-Dobbe JC, Liu R, Tong G, et al (2012) Arterivirus minor envelope proteins are a major determinant of viral tropism in cell culture. J Virol 86: 3701–3712. 22258262 10.1128/JVI.06836-11 PMC3302522

[6] Lu Z, Zhang J, Huang CM, Go YY, Faaberg KS, et al (2012) Chimeric viruses containing the N-terminal ectodomains of GP5 and M proteins of porcine reproductive and respiratory syndrome virus do not change the cellular tropism of equine arteritis virus. Virology 432: 99–109. 22739441 10.1016/j.virol.2012.05.022

[7] Das PB, Dinh PX, Ansari IH, de Lima M, Osorio FA, et al (2010) The minor envelope glycoproteins GP2a and GP4 of porcine reproductive and respiratory syndrome virus interact with the receptor CD163. J Virol 84: 1731–1740. 19939927 10.1128/JVI.01774-09 PMC2812361

[8] Snijder EJ, Dobbe JC, Spaan WJ (2003) Heterodimerization of the two major envelope proteins is essential for arterivirus infectivity. J Virol 77: 97–104. 12477814 10.1128/JVI.77.1.97-104.2003 PMC140607

[9] Johnson CR, Griggs TF, Gnanandarajah J, Murtaugh MP (2011) Novel structural protein in porcine reproductive and respiratory syndrome virus encoded by an alternative ORF5 present in all arteriviruses. J Gen Virol 92: 1107–1116. 21307222 10.1099/vir.0.030213-0 PMC3139420

[10] Firth AE, Zevenhoven-Dobbe JC, Wills NM, Go YY, Balasuriya UB, et al (2011) Discovery of a small arterivirus gene that overlaps the GP5 coding sequence and is important for virus production. J Gen Virol 92: 1097–1106. 21307223 10.1099/vir.0.029264-0 PMC3139419

[11] Wissink EH, Kroese MV, van Wijk HA, Rijsewijk FA, Meulenberg JJ, et al (2005) Envelope protein requirements for the assembly of infectious virions of porcine reproductive and respiratory syndrome virus. J Virol 79: 12495–12506. 16160177 10.1128/JVI.79.19.12495-12506.2005 PMC1211556

[12] Van Breedam W, Van Gorp H, Zhang JQ, Crocker PR, Delputte PL, et al (2010) The M/GP(5) glycoprotein complex of porcine reproductive and respiratory syndrome virus binds the sialoadhesin receptor in a sialic acid-dependent manner. PLoS Pathog 6: e1000730. 20084110 10.1371/journal.ppat.1000730 PMC2799551

[13] Thanawongnuwech R, Halbur PG, Thacker EL (2000) The role of pulmonary intravascular macrophages in porcine reproductive and respiratory syndrome virus infection. Anim Health Res Rev 1: 95–102. 11708601 10.1017/s1466252300000086

[14] Done SH, Paton DJ, White ME (1996) Porcine reproductive and respiratory syndrome (PRRS): a review, with emphasis on pathological, virological and diagnostic aspects. Br Vet J 152: 153–174. 8680839 10.1016/S0007-1935(96)80071-6 PMC7130409

[15] Chand RJ, Trible BR, Rowland RR (2012) Pathogenesis of porcine reproductive and respiratory syndrome virus. Curr Opin Virol 2: 256–263. 22709514 10.1016/j.coviro.2012.02.002

[16] Allende R, Laegreid WW, Kutish GF, Galeota JA, Wills RW, et al (2000) Porcine reproductive and respiratory syndrome virus: description of persistence in individual pigs upon experimental infection. J Virol 74: 10834–10837. 11044133 10.1128/jvi.74.22.10834-10837.2000 PMC110963

[17] Wills RW, Doster AR, Galeota JA, Sur JH, Osorio FA (2003) Duration of infection and proportion of pigs persistently infected with porcine reproductive and respiratory syndrome virus. J Clin Microbiol 41: 58–62. 12517825 10.1128/JCM.41.1.58-62.2003 PMC149563

[18] Mateu E, Diaz I (2008) The challenge of PRRS immunology. Vet J 177: 345–351. 17644436 10.1016/j.tvjl.2007.05.022 PMC7110845

[19] Yoon IJ, Joo HS, Goyal SM, Molitor TW (1994) A modified serum neutralization test for the detection of antibody to porcine reproductive and respiratory syndrome virus in swine sera. J Vet Diagn Invest 6: 289–292. 7948196 10.1177/104063879400600326

[20] Pirzadeh B, Dea S (1997) Monoclonal antibodies to the ORF5 product of porcine reproductive and respiratory syndrome virus define linear neutralizing determinants. J Gen Virol 78: 1867–1873. 9266981 10.1099/0022-1317-78-8-1867

[21] Gonin P, Pirzadeh B, Gagnon CA, Dea S (1999) Seroneutralization of porcine reproductive and respiratory syndrome virus correlates with antibody response to the GP5 major envelope glycoprotein. J Vet Diagn Invest 11: 20–26. 9925207 10.1177/104063879901100103

[22] Li J, Murtaugh MP (2012) Dissociation of porcine reproductive and respiratory syndrome virus neutralization from antibodies specific to major envelope protein surface epitopes. Virology 433: 367–376. 22981434 10.1016/j.virol.2012.08.026

[23] Yang L, Frey ML, Yoon KJ, Zimmerman JJ, Platt KB (2000) Categorization of North American porcine reproductive and respiratory syndrome viruses: epitopic profiles of the N, M, GP5 and GP3 proteins and susceptibility to neutralization. Arch Virol 145: 1599–1619. 11003472 10.1007/s007050070079

[24] Vanhee M, Costers S, Van Breedam W, Geldhof MF, Van Doorsselaere J, et al (2010) A variable region in GP4 of European-type porcine reproductive and respiratory syndrome virus induces neutralizing antibodies against homologous but not heterologous virus strains. Viral Immunol 23: 403–413. 20712485 10.1089/vim.2010.0025 PMC2928701

[25] Osorio FA, Galeota JA, Nelson E, Brodersen B, Doster A, et al (2002) Passive transfer of virus-specific antibodies confers protection against reproductive failure induced by a virulent strain of porcine reproductive and respiratory syndrome virus and establishes sterilizing immunity. Virology 302: 9–20. 12429512 10.1006/viro.2002.1612

[26] Lopez OJ, Oliveira MF, Garcia EA, Kwon BJ, Doster A, et al (2007) Protection against porcine reproductive and respiratory syndrome virus (PRRSV) infection through passive transfer of PRRSV-neutralizing antibodies is dose dependent. Clin Vaccine Immunol 14: 269–275. 17215336 10.1128/CVI.00304-06 PMC1828847

[27] Kwon B, Ansari IH, Pattnaik AK, Osorio FA (2008) Identification of virulence determinants of porcine reproductive and respiratory syndrome virus through construction of chimeric clones. Virology 380: 371–378. 18768197 10.1016/j.virol.2008.07.030

[28] Chang CC, Yoon KJ, Zimmerman JJ, Harmon KM, Dixon PM, et al (2002) Evolution of porcine reproductive and respiratory syndrome virus during sequential passages in pigs. J Virol 76: 4750–4763. 11967292 10.1128/JVI.76.10.4750-4763.2002 PMC136148

[29] Rowland RR, Steffen M, Ackerman T, Benfield DA (1999) The evolution of porcine reproductive and respiratory syndrome virus: quasispecies and emergence of a virus subpopulation during infection of pigs with VR-2332. Virology 259: 262–266. 10388650 10.1006/viro.1999.9789

[30] Kimman TG, Cornelissen LA, Moormann RJ, Rebel JM, Stockhofe-Zurwieden N (2009) Challenges for porcine reproductive and respiratory syndrome virus (PRRSV) vaccinology. Vaccine 27: 3704–3718. 19464553 10.1016/j.vaccine.2009.04.022

[31] Murtaugh MP, Genzow M (2011) Immunological solutions for treatment and prevention of porcine reproductive and respiratory syndrome (PRRS). Vaccine 29: 8192–8204. 21925560 10.1016/j.vaccine.2011.09.013

[32] Lopez OJ, Osorio FA (2004) Role of neutralizing antibodies in PRRSV protective immunity. Vet Immunol Immunopathol 102: 155–163. 15507302 10.1016/j.vetimm.2004.09.005

[33] Plagemann PG, Rowland RR, Faaberg KS (2002) The primary neutralization epitope of porcine respiratory and reproductive syndrome virus strain VR-2332 is located in the middle of the GP5 ectodomain. Arch Virol 147: 2327–2347. 12491101 10.1007/s00705-002-0887-2

[34] Ostrowski M, Galeota JA, Jar AM, Platt KB, Osorio FA, et al (2002) Identification of neutralizing and nonneutralizing epitopes in the porcine reproductive and respiratory syndrome virus GP5 ectodomain. J Virol 76: 4241–4250. 11932389 10.1128/JVI.76.9.4241-4250.2002 PMC155073

[35] Cleveland SM, Buratti E, Jones TD, North P, Baralle F, et al (2000) Immunogenic and antigenic dominance of a nonneutralizing epitope over a highly conserved neutralizing epitope in the gp41 envelope glycoprotein of human immunodeficiency virus type 1: its deletion leads to a strong neutralizing response. Virology 266: 66–78. 10612661 10.1006/viro.1999.0041

[36] Wissink EH, van Wijk HA, Kroese MV, Weiland E, Meulenberg JJ, et al (2003) The major envelope protein, GP5, of a European porcine reproductive and respiratory syndrome virus contains a neutralization epitope in its N-terminal ectodomain. J Gen Virol 84: 1535–1543. 12771423 10.1099/vir.0.18957-0

[37] von Heijne G (1983) Patterns of amino acids near signal-sequence cleavage sites. Eur J Biochem 133: 17–21. 6852022 10.1111/j.1432-1033.1983.tb07424.x

[38] Chavan M, Lennarz W (2006) The molecular basis of coupling of translocation and N-glycosylation. Trends Biochem Sci 31: 17–20. 16356726 10.1016/j.tibs.2005.11.010

[39] Petersen TN, Brunak S, von Heijne G, Nielsen H (2011) SignalP 4.0: discriminating signal peptides from transmembrane regions. Nat Methods 8: 785–786. 21959131 10.1038/nmeth.1701

[40] Blobel G, Dobberstein B (1975) Transfer of proteins across membranes. II. Reconstitution of functional rough microsomes from heterologous components. J Cell Biol 67: 852–862. 811672 10.1083/jcb.67.3.852 PMC2111655

[41] Delisle B, Gagnon CA, Lambert ME, D’Allaire S (2012) Porcine reproductive and respiratory syndrome virus diversity of Eastern Canada swine herds in a large sequence dataset reveals two hypervariable regions under positive selection. Infect Genet Evol 12: 1111–1119. 22484762 10.1016/j.meegid.2012.03.015

[42] Kornfeld R, Kornfeld S (1985) Assembly of asparagine-linked oligosaccharides. Annu Rev Biochem 54: 631–664. 3896128 10.1146/annurev.bi.54.070185.003215

[43] Kim HS, Kwang J, Yoon IJ, Joo HS, Frey ML (1993) Enhanced replication of porcine reproductive and respiratory syndrome (PRRS) virus in a homogeneous subpopulation of MA-104 cell line. Arch Virol 133: 477–483. 8257302 10.1007/BF01313785

[44] Ansari IH, Kwon B, Osorio FA, Pattnaik AK (2006) Influence of N-linked glycosylation of porcine reproductive and respiratory syndrome virus GP5 on virus infectivity, antigenicity, and ability to induce neutralizing antibodies. J Virol 80: 3994–4004. 16571816 10.1128/JVI.80.8.3994-4004.2006 PMC1440468

[45] Cooper VL, Doster AR, Hesse RA, Harris NB (1995) Porcine reproductive and respiratory syndrome: NEB-1 PRRSV infection did not potentiate bacterial pathogens. J Vet Diagn Invest 7: 313–320. 7578444 10.1177/104063879500700303

[46] Allende R, Kutish GF, Laegreid W, Lu Z, Lewis TL, et al (2000) Mutations in the genome of porcine reproductive and respiratory syndrome virus responsible for the attenuation phenotype. Arch Virol 145: 1149–1161. 10948988 10.1007/s007050070115 PMC7086797

[47] Cheung JC, Reithmeier RA (2007) Scanning N-glycosylation mutagenesis of membrane proteins. Methods 41: 451–459. 17367717 10.1016/j.ymeth.2006.10.002

[48] Vu HL, Kwon B, Yoon KJ, Laegreid WW, Pattnaik AK, et al (2011) Immune evasion of porcine reproductive and respiratory syndrome virus through glycan shielding involves both glycoprotein 5 as well as glycoprotein 3. J Virol 85: 5555–5564. 21411530 10.1128/JVI.00189-11 PMC3094951

[49] Faaberg KS, Hocker JD, Erdman MM, Harris DL, Nelson EA, et al (2006) Neutralizing antibody responses of pigs infected with natural GP5 N-glycan mutants of porcine reproductive and respiratory syndrome virus. Viral Immunol 19: 294–304. 16817772 10.1089/vim.2006.19.294

[50] Ephraim E, Burwinkel M, Palissa C, Wei Z, Simon O, et al.. (2009) Establishment of new porcine intestinal epithelial cell lines for the study of anti-viral effects of probiotics. European Veterinary Immunology Workshop (EVIW 2009). Berlin, Germany. 45.

[51] Green MR, Sambrook J (2012) Molecular Cloning: A Laboratory Manual. 4th edition. Cold Spring Harbor, NY, USA: Cold Spring Harbor Laboratory Press.

[52] Bieniossek C, Imasaki T, Takagi Y, Berger I (2012) MultiBac: expanding the research toolbox for multiprotein complexes. Trends Biochem Sci 37: 49–57. 22154230 10.1016/j.tibs.2011.10.005 PMC7127121

[53] Han J, Liu G, Wang Y, Faaberg KS (2007) Identification of nonessential regions of the nsp2 replicase protein of porcine reproductive and respiratory syndrome virus strain VR-2332 for replication in cell culture. J Virol 81: 9878–9890. 17522233 10.1128/JVI.00562-07 PMC2045381

[54] Veit M, Ponimaskin E, Schmidt MFG (2002) Analysis of S-acylation of proteins. Methods Mol Biol 194: 159–178. 12029833 10.1385/1-59259-181-7:159

[55] Walter P, Blobel G (1983) Preparation of microsomal membranes for cotranslational protein translocation. Methods Enzymol 96: 84–93. 6656655 10.1016/s0076-6879(83)96010-x

[56] Lee YJ, Park CK, Nam E, Kim SH, Lee OS, et al (2010) Generation of a porcine alveolar macrophage cell line for the growth of porcine reproductive and respiratory syndrome virus. J Virol Methods 163: 410–415. 19900480 10.1016/j.jviromet.2009.11.003

[57] Faaberg KS, Plagemann PG (1995) The envelope proteins of lactate dehydrogenase-elevating virus and their membrane topography. Virology 212: 512–525. 7571421 10.1006/viro.1995.1509

