# Supplementary material for: Signal Peptide Cleavage from GP5 of PRRSV: A Minor Fraction of Molecules Retains the Decoy Epitope, a Presumed Molecular Cause for Viral Persistence
Source: PLoS One. 2013 Jun 6;8(6):e65548. doi: 10.1371/journal.pone.0065548 (PMC3675037; doi:10.1371/journal.pone.0065548)
Supplement: Table S1 — Amino acid sequences and signal peptide cleavage site prediction for the GP5 sequences analyzed in this study. For all GP5 variants under study, the sequence of residues 20–40 is listed along with parameters from signal peptide cleavage prediction using SignalP 4.0 (www.cbs.dtu.dk/services/SignalP/): D score (likelihood of signal peptide cleavage immediately before the site given in brackets), and Y scores for “site 1” (A26|V27) and “site 2” (A31|V32). The higher Y, the more likely signal peptide cleavage at this site. Values above 0.5 can be considered “above the threshold”. Most probable cleavage site is indicated by Y score in bold. – In the sequence, the residues in the predicted signal peptide are in small letters, the residues in the mature protein in capital letters. Potential glycosylation sites according to the sequon NXS/T are annotated in bold, the “decoy epitope” sequence (VLAN) is in italics in the GP5 sequence from VR-2332 wt. – GenBank references: VR-2332 [AAD12129.1], MLV RespPRRS [AAD27656.1], JXA-1 [ABL60902.1], Neb-1 [ACE87854.1]. Full-length protein sequences were submitted to the SignalP 4.0 prediction. (DOCX) [file pone.0065548.s001.docx]

**Table S1:** Amino acid sequences and signal peptide cleavage site prediction for the GP5 sequences analyzed in this study.

| **Strain** | **Sequence (residues 20–40)** | **D score at cleav. site** | **Y score for A26\|V27** | **Y score for A31\|S32** |
| --- | --- | --- | --- | --- |
| **VR-2332 wt** | vpfcfa*vla****n***aS**N**DSSSHLQ | 0.763 (S32) | 0.667 | **0.720** |
| **VR-2332 N30S** | vpfcfavlasaS**N**DSSSHLQ | 0.783 (S32) | 0.652 | **0.730** |
| **VR-2332 N33S** | vpfcfavla**n**aSSDSSSHLQ | 0.754 (S32) | 0.668 | **0.688** |
| **VR-2332 N30/33S** | vpfcfavlasaSSDSSSHLQ | 0.772 (S32) | 0.653 | **0.694** |
| **VR-2332 D34N** | vpfcfavla**n**aS**NN**SSSHLQ | 0.766 (S32) | 0.665 | **0.733** |
| **VR-2332 uncl.** | VPFCFFVLY**N**FS**N**DSSSHLQ | (0.295) | 0.186 | 0.170 |
| **VR-2332 cl.1** | vpfcfaVLS**N**YS**N**DSSSHLQ | 0.754 (V27) | **0.707** | 0.298 |
| **VR-2332 cl.2** | vpfcffvls**n**aS**N**DSSSHLQ | 0.735 (S32) | 0.338 | **0.684** |
| **MLV RespPRRS** | vpfcfaVLA**N**AS**N**DSSSHLQ | 0.755 (V27) | **0.703** | 0.683 |
| **JXA-1** | vpfylavlv**n**aSN**NN**SSHIQ | 0.875 (S32) | 0.703 | **0.835** |
| **Neb-1** | vpfcfaVLV**N**ASYSSSSHLQ | 0.525 (V27) | **0.547** | 0.514 |

For all GP5 variants under study, the sequence of residues 20–40 is listed along with parameters from signal peptide cleavage prediction using SignalP 4.0 ([www.cbs.dtu.dk/services/](http://www.cbs.dtu.dk/services/SignalP/)**[SignalP](http://www.cbs.dtu.dk/services/SignalP/)**[/](http://www.cbs.dtu.dk/services/SignalP/)): D score (likelihood of signal peptide cleavage immediately before the site given in brackets), and Y scores for “site 1” (A26|V27) and “site 2” (A31|V32). The higher Y, the more likely signal peptide cleavage at this site. Values above 0.5 can be considered “above the threshold”. Most probable cleavage site is indicated by Y score in **bold**. – In the sequence, the residues in the predicted signal peptide are in small letters, the residues in the mature protein in capital letters. Potential glycosylation sites according to the sequon **N**XS/T are annotated in bold, the “decoy epitope” sequence (*VLAN*) is in italics in the GP5 sequence from VR-2332 wt. – GenBank references: VR-2332 [AAD12129.1], MLV RespPRRS [AAD27656.1], JXA-1 [ABL60902.1], Neb-1 [ACE87854.1]. Full-length protein sequences were submitted to the SignalP 4.0 prediction.
