# Supplementary material for: Signal Peptide Cleavage from GP5 of PRRSV: A Minor Fraction of Molecules Retains the Decoy Epitope, a Presumed Molecular Cause for Viral Persistence
Source: PLoS One. 2013 Jun 6;8(6):e65548. doi: 10.1371/journal.pone.0065548 (PMC3675037; doi:10.1371/journal.pone.0065548)
Supplement: Table S2 — Chymotryptic peptides identified by mass spectrometry from virus-derived GP5 and from recombinantly expressed GP5. Representative list of ectodomain peptides of deglycosylated GP5, digested with chymotrypsin, that were identified by mass spectrometry (LC-MS/MS), source: enriched PRRS virus particles and recombinant protein expression of GP5–M in Sf9 insect cells (Baculovirus system). Sequences of peptides along with their observed and calculated masses, number of missed chymotrypsin cleavage sites, and their ions score (as a measure of confidence of the mass match and taken from the MASCOT program). Note that glycosylated asparagine (N) is converted to aspartic acid (D) by deglycosylation with PNGase F. Peptides starting with V27 as well as with S32 were identified in both types of sample, which is evidence for use of signal peptide cleavage sites 1 (A26|V27) and 2 (A31|S32). – * The sequence between the dots indicates the peptide which was identified by MS/MS. (DOCX) [file pone.0065548.s002.docx]

**Supplemental Table 2:** Chymotryptic peptides identified by mass spectrometry from virus-derived GP5 and from recombinantly expressed GP5.

| **Start** | **End** | **Observed** | **Mr(calc)** | **Miss** | **Sequence*** | **Ions Score** |
| --- | --- | --- | --- | --- | --- | --- |
| **GP5 from virus** | | | | | | |
| 27 | 40 | 657.82 | 1313.62 | 1 | -.VLANASNDSSSHL.Q | 69 |
| 27 | 40 | 658.31 | 1314.61 | 1 | -.VLANASDDSSSHL.Q | 43 |
| 27 | 40 | 658.80 | 1315.59 | 1 | -.VLADASDDSSSHL.Q | 46 |
| 27 | 42 | 778.88 | 1555.75 | 2 | -.VLANASDDSSSHLQL.I | 48 |
| 27 | 42 | 779.37 | 1556.73 | 2 | -.VLADASDDSSSHLQL.I | 58 |
| 27 | 44 | 916.96 | 1831.90 | 3 | -.VLANASDDSSSHLQLIY.D | 64 |
| 27 | 44 | 917.45 | 1832.88 | 3 | -.VLADASDDSSSHLQLIY.D | 53 |
| 28 | 40 | 552.23 | 1102.45 | 0 | L.ANASDDSSSHL.Q | 28 |
| 28 | 44 | 810.88 | 1619.74 | 2 | L.ANASDDSSSHLQLIY.D | 33 |
| 28 | 44 | 811.37 | 1620.73 | 2 | L.ADASDDSSSHLQLIY.D | 32 |
| 32 | 42 | 544.26 | 1086.49 | 1 | -.SNDSSSHLQL.I | 27 |
| 32 | 42 | 544.75 | 1087.48 | 1 | -.SDDSSSHLQL.I | 52 |
| 32 | 44 | 682.33 | 1362.64 | 2 | -.SNDSSSHLQLIY.N | 52 |
| 32 | 44 | 682.82 | 1363.63 | 2 | -.SDDSSSHLQLIY.D | 42 |
| 32 | 46 | 796.88 | 1591.74 | 3 | -.SDDSSSHLQLIYDL.T | 51 |
| 32 | 48 | 903.94 | 1805.87 | 4 | -.SDDSSSHLQLIYDLTL.C | 26 |
| 32 | 56 | 1399.13 | 2796.24 | 6 | -.SDDSSSHLQLIYDLTLCELDGTDW.L Propionamide (C) | 45 |
| 41 | 61 | 1151.06 | 2300.10 | 6 | L.IYDLTLCELDGTDWLANKF.D Propionamide (C) | 87 |
| 41 | 56 | 726.32 | 1450.63 | 3 | Y.DLTLCELDGTDW.L Propionamide (C) | 71 |
| 43 | 56 | 1012.99 | 2023.96 | 5 | Y.DLTLCELDGTDWLANKF.D Propionamide (C) | 84 |
| 50 | 56 | 583.78 | 1165.54 | 2 | L.DGTDWLANKF.D | 50 |
| **Recombinant GP5 (Baculovirus/insect cells)** | | | | | | |
| 27 | 44 | 916.96 | 1831.90 | 3 | -.VLANASDDSSSHLQLIY.D | 53 |
| 27 | 44 | 917.45 | 1832.88 | 3 | -.VLADASDDSSSHLQLIY.D | 59 |
| 28 | 44 | 810.39 | 1618.76 | 2 | L.ANASNDSSSHLQLIY.N | 46 |
| 32 | 42 | 544.26 | 1086.49 | 1 | -.SNDSSSHLQL.I | 33 |
| 32 | 42 | 544.75 | 1087.48 | 1 | -.SDDSSSHLQL.I | 45 |
| 32 | 44 | 682.33 | 1362.64 | 2 | -.SNDSSSHLQLIY.N | 56 |
| 32 | 44 | 682.82 | 1363.63 | 2 | -.SDDSSSHLQLIY.D | 41 |

Representative list of ectodomain peptides of deglycosylated GP5, digested with chymotrypsin, that were identified by mass spectrometry (LC-MS/MS), source: enriched PRRS virus particles and recombinant protein expression of GP5–M in *Sf*9 insect cells (Baculovirus system). Sequences of peptides along with their observed and calculated masses, number of missed chymotrypsin cleavage sites, and their ions score (as a measure of confidence of the mass match and taken from the MASCOT program). Note that glycosylated asparagine (N) is converted to aspartic acid (D) by deglycosylation with PNGase F. Peptides starting with V27 as well as with S32 were identified in both types of sample, which is evidence for use of signal peptide cleavage sites 1 (A26|V27) and 2 (A31|S32). – * The sequence between the dots indicates the peptide which was identified by MS/MS.
